# Manipulation of the Xanthophyll Cycle Increases Plant Susceptibility to *Sclerotinia sclerotiorum*

**DOI:** 10.1371/journal.ppat.1004878

**Published:** 2015-05-20

**Authors:** Jun Zhou, Lizhang Zeng, Jian Liu, Da Xing

**Affiliations:** MOE Key Laboratory of Laser Life Science & Institute of Laser Life Science, College of Biophotonics, South China Normal University, Guangzhou, China; Wageningen University, Netherlands

## Abstract

The xanthophyll cycle is involved in dissipating excess light energy to protect the photosynthetic apparatus in a process commonly assessed from non-photochemical quenching (NPQ) of chlorophyll fluorescence. Here, it is shown that the xanthophyll cycle is modulated by the necrotrophic pathogen *Sclerotinia sclerotiorum* at the early stage of infection. Incubation of *Sclerotinia* led to a localized increase in NPQ even at low light intensity. Further studies showed that this abnormal change in NPQ was closely correlated with a decreased pH caused by *Sclerotinia*-secreted oxalate, which might decrease the ATP synthase activity and lead to a deepening of thylakoid lumen acidification under continuous illumination. Furthermore, suppression (with dithiothreitol) or a defect (in the *npq1-2* mutant) of violaxanthin de-epoxidase (VDE) abolished the *Sclerotinia*-induced NPQ increase. HPLC analysis showed that the *Sclerotinia*-inoculated tissue accumulated substantial quantities of zeaxanthin at the expense of violaxanthin, with a corresponding decrease in neoxanthin content. Immunoassays revealed that the decrease in these xanthophyll precursors reduced *de novo* abscisic acid (ABA) biosynthesis and apparently weakened tissue defense responses, including ROS induction and callose deposition, resulting in enhanced plant susceptibility to *Sclerotinia*. We thus propose that *Sclerotinia* antagonizes ABA biosynthesis to suppress host defense by manipulating the xanthophyll cycle in early pathogenesis. These findings provide a model of how photoprotective metabolites integrate into the defense responses, and expand the current knowledge of early plant-*Sclerotinia* interactions at infection sites.

## Author Summary

In recent years, the role of the chloroplast in the defense against microbes has been intensively investigated and is of high interest to both plant-microbe interaction and photosynthesis research. The xanthophyll cycle is well known to be involved in dissipating excess light energy to protect the photosynthetic apparatus in a process commonly assessed via non-photochemical quenching (NPQ) of chlorophyll fluorescence. Recent studies show that NPQ can be positively or negatively affected by pathogen attack. However, knowledge about the regulatory processes by which pathogens affect NPQ, as well as their impact on plant defense responses, is incomplete. This work characterized the impact of infection of *Arabidopsis* leaves by the necrotrophic pathogen *Sclerotinia sclerotiorum* on the xanthophyll cycle. Our research revealed for the first time that *Sclerotinia* uses a novel strategy involving manipulation of the xanthophyll cycle to weaken host defense responses and increase its successful colonization of host cells. These findings contribute to understanding the plant-*Sclerotinia* interactions in early pathogenesis, which will provide new sights into the development of strategies to increase *Sclerotinia* resistance in plants for practical applications.

## Introduction

Chloroplasts are not only the factory for photosynthesis, but are also involved in various types of plant-pathogen interactions [1–3]. Indeed, the process of photosynthesis is functionally linked to plant immunity by providing energy, reducing equivalents and carbon skeletons [4–9] as well as producing oxidants and oxidant-derived hormonal messengers with roles in defense responses [10–11]. Light energy absorbed by the harvesting antenna complexes is transferred to reaction centers to drive photochemistry. However, when the rate of excitation energy exceeds the capacity for light utilization, excited-state chlorophyll can be de-excited by thermal dissipation in a process that is commonly assessed as non-photochemical quenching (NPQ) of chlorophyll fluorescence [12–15]. Mechanisms involved in thermal energy dissipation include the xanthophylls zeaxanthin and lutein, the photosystem II subunit S (PsbS) protein, as well as energetic couplings between the core antenna complexes and LHCII [16–23]. The most rapid component of NPQ is called qE, which is activated by a decrease in thylakoid lumen pH [13,15,24–25]. In the xanthophyll cycle, low pH activates violaxanthin de-epoxidase (VDE) that converts violaxanthin into zeaxanthin via the intermediate antheraxanthin. Conversely, under low light and relatively alkaline conditions, zeaxanthin epoxidase (ZEP) catalyzes conversion of zeaxanthin via antheraxanthin into violaxanthin, thus forming an integrated cycle [26]. While there is a school of thought that addressed the zeaxanthin and PsbS-dependent qE as separate mechanisms, the elegant works by Demmig-Adams & Adams group have proposed that these are two parts of the same process, where the xanthophyll cycle generates zeaxanthin, and PsbS triggers the actual engagement of zeaxanthin in thermal dissipation [12, 27].

At present, although the xanthophyll cycle is well known to be involved in photoprotection, it has not been as deeply characterized in plant disease responses. Several recent studies, however, have shown that there is a correlation between NPQ changes and resistance to pathogens [28–32]. The deletion of PsbS in the *npq4-1* mutant was shown to alter jasmonate metabolism and render plant less attractive for herbivores [28–29]. Moreover, NPQ formation is negatively correlated with reactive oxygen species (ROS) production under excess light [11,15,33], and weakening NPQ may promote ^1^O_2_ generation in PSII [26,33]. In particular, in the *PsbS/vde1* double mutant, treatment with flg22 enhances ROS production and early defense marker gene expression [30]. In addition, the intensity of NPQ was also positively or negatively affected by various pathogen attacks, increasing around the infected regions but decreasing in its core [34–35]. This variability in NPQ might depend on the degree of tissue damage [35]. However, knowledge about the regulatory processes of pathogens on NPQ as well as their impact on plant defense responses is incomplete.

The xanthophyll precursor pool plays an important role in the biosynthesis of the phytohormone abscisic acid (ABA) [36–38]. *De novo* synthesis of ABA requires ZEP-catalyzed epoxidation of zeaxanthin to violaxanthin. Subsequently, the violaxanthin-derivatives neoxanthin and xanthoxin are converted into ABA through a series of isomerization and dehydrogenation reactions [39]. In the ABA-deficient mutant *aba1* (an allele of *npq2*), ZEP is not functional, causes accumulation of zeaxanthinin parallel with decreases in the epoxy-xanthophylls antheraxanthin, violaxanthin and neoxanthin [40–41]. In the xanthophyll cycle, VDE requires ascorbate as a reductant to convert violaxanthin to zeaxanthin [42]. As a result, reduced levels of ascorbate in the *Arabidopsis vtc1* (vitamin C1) mutant stimulate ABA production [43]. In contrast, enhanced VDE activity can reduce ascorbate levels and antagonize ABA synthesis [43]. Thus, the regulation of the xanthophyll cycle allows ABA levels to be modified, which could be a subtle mechanism exploited by pathogens to lower plant resistance.

NPQ is regulated on a fast timescale by changes in thylakoid lumen pH [15,19,44]. Disruption of the pH gradient (ΔpH) across the thylakoid membrane with the ionophore nigericin can abolish NPQ formation [45]. Conversely, NPQ can be induced in isolated thylakoids by lowering ambient pH [46,47]. In the pathogenesis of pathogenic fungi, local pH can be dynamically altered by the pathogen as host colonization advances [48]. In fact, pH value is one of the major features affecting maximal activity of pathogenicity factors, such as the arsenal of cell wall degradative enzymes that display acidic pH-specific expression in the necrotrophic pathogen *Sclerotinia sclerotiorum* [49,50]. *Sclerotinia* decreases host pH by secreting millimolar quantities of oxalate [51,52]. Oxalate exhibits versatile functions in plant infection and fungal development [53]; it triggers plant programmed cell death [54–56], suppresses plant oxidative burst and callose deposition [57–59], and inhibits ABA-induced stomatal closure [60]. Functional genetic studies have provided evidence for the relevance of ABA in plant defense against *Sclerotinia* [61–63]. However, it is still unknown whether the ambient pH changes would affect the xanthophyll cycle and subsequent ABA biosynthesis in the pathogenesis of *Sclerotinia*.

Here, we investigated the interplay of the xanthophyll cycle and plant resistance to the necrotrophic pathogen *Sclerotinia*. The results show that *Sclerotinia* caused a dysfunction of the xanthophyll cycle during initial stages of infection, with leaves displaying an abnormal increase in NPQ in a zeaxanthin-related manner even under low light conditions. Further studies revealed that decreases in the precursor violaxanthin were associated with limited ABA biosynthesis, which, in turn, apparently weakened tissue defense responses and eased *Sclerotinia* colonization of the host plant. These findings present a mechanism of how photoprotective metabolites integrate into the defense work and contribute to understanding the early plant-*Sclerotinia* interactions at the infection site.

## Results

### *Sclerotinia* infection leads to a localized increase in NPQ

**Fig 1:**
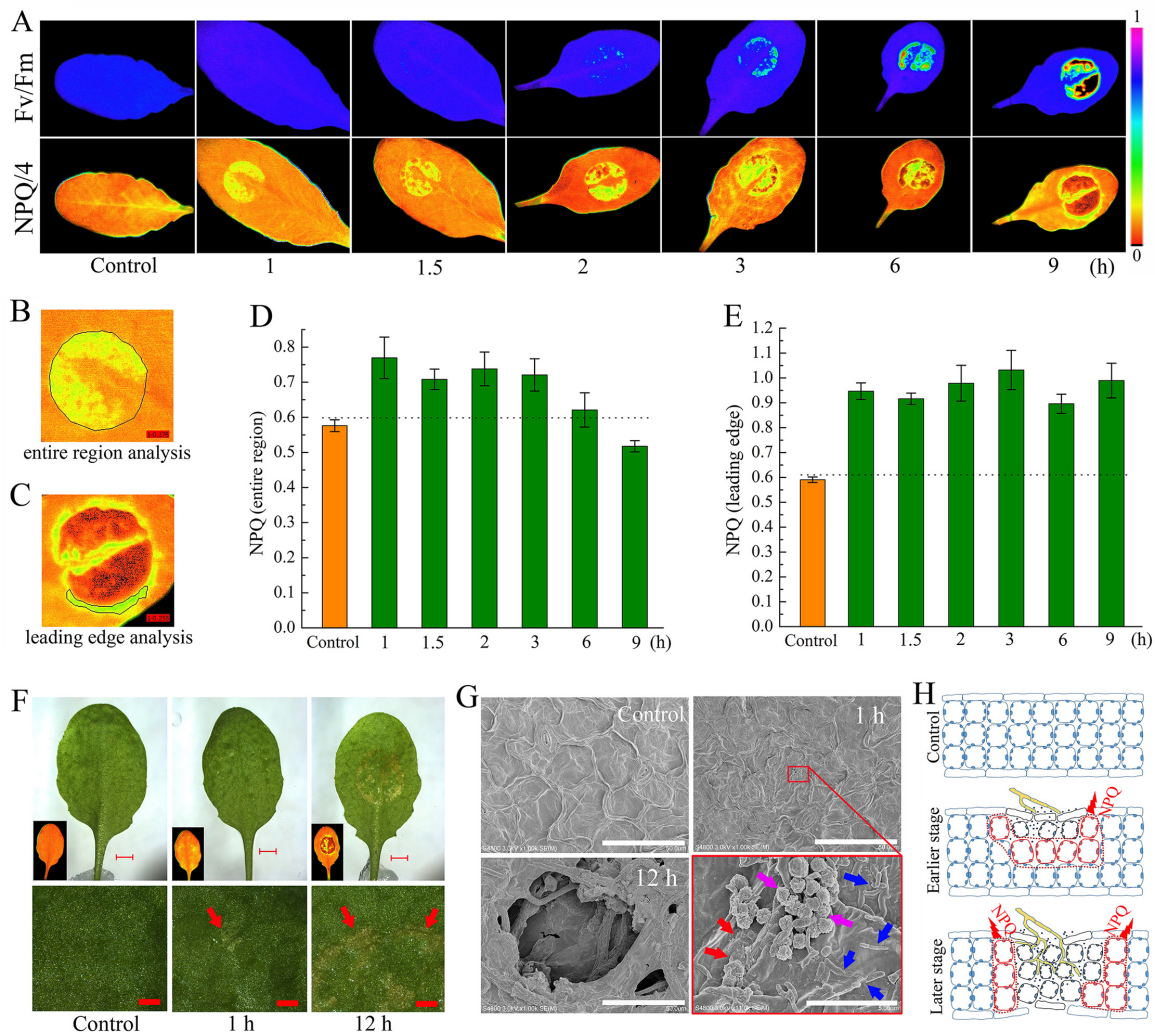
Effect of *Sclerotinia* infection on NPQ. (A) Chlorophyll fluorescence imaging showing changes in Fv/Fm and NPQ in *Sclerotinia*-infected *Arabidopsis* leaves. After inoculation with *Sclerotinia*, plants were dark-adapted for 1 h prior to measurement of chlorophyll fluorescence. Because NPQ values always higher than one but rarely exceeded four, NPQ/4 was displayed by pseudo-color with values ranging from zero (black) to one (purple). (B, C) Examples for statistical analysis of NPQ in the entire inoculated region (B) and the leading edge (C). (D, E) Quantitative analysis of the values of NPQ in *Sclerotinia*-infected entire region (D) and leading edge (E). Values are means ± _SE_ of three replicates. (F) Infection phenotypes that related NPQ changes after inoculated with *Sclerotinia*. Inset: the corresponding NPQ/4 image. Arrows indicate the water soaking phenotypes. Scale bars in original (upper) and magnified (below) pictures are 2 mm and 0.5 mm, respectively. (G) Scanning electron micrographs showing detailed changes on the leaf surface after inoculated with *Sclerotinia*. Pictures were obtained with a cold field scanning electron microscope at an accelerating voltage of 3.0 kV. Red arrows indicate the slight broken cuticle; Blue arrows indicate mycelial cells; Purple arrows indicate amorphous structures that might be derived from PDA plugs. Scale bars in original and magnified images are 50 μm and 5 μm, respectively. (H) A model to explain NPQ variation at different infection stages and regions. The growing hyphae are painted with yellow color. Drawing of plant cells with blue lines represent un-infected zone; red lines represent leading edge of infected zone; dark lines meant severely damaged tissue.

When analyzing the timing and spread of *Sclerotinia* in its host plant with chlorophyll fluorescence imaging, we identified anomalies of NPQ in plant tissue during early infection. Fig 1A shows images of two conventional fluorescence parameters, Fv/Fm (maximum photochemical efficiency of PSII) and NPQ, in randomly selected *Arabidopsis* leaves. *Sclerotinia* infection induced a gradual decrease in Fv/Fm, which indirectly reflected the degree of tissue damage [64]. Interestingly, a localized increase in NPQ was observed already 1 h after infection. As inoculation time prolonged, NPQ decreased in the core of the necrotic lesions but increased around the necrosis. Because the infected areas did not behave homogeneously, possibly due to contact spot variances on the uneven foliage, the entire inoculated region and the leading edge were selected for statistical analysis, respectively (Fig 1B and 1C). Although the mean values of NPQ decreased significantly in severely damaged tissue (as shown at 9 h), NPQ remained at high levels from 1 to 3 h after infection (Fig 1D). The increase in NPQ was most pronounced at the leading edge (Fig 1E).

Next, changes in NPQ within the context of the penetration of the host by *Sclerotinia* were assessed. A water-soaked appearance began to emerge at 1 h but exhibited severely at 12 h after infection (Fig 1F). The earlier slight damage might be caused by oxalate in the PDA plug. At early stage, a number of scattered mycelial cells on the leaf surface were observed under scanning electron microscope (Fig 1G). Further results revealed that the infection cushions began forming at 8 h and hyphae were interweaved in the necrotic tissue at later stage (Figs 1G and S1). These features suggest that the *Sclerotinia*-induced NPQ increase is an early event that occurs prior to infection cushions formation. A model is proposed to depict how NPQ is related to the infection process (Fig 1H): During the early stage, upper-side cell damage causes a slight decrease in Fv/Fm but greatly enhances NPQ throughout the entire region. As inoculated tissue moves toward necrosis, it exhibits increased NPQ in the leading edge, whereas both Fv/Fm and NPQ are reduced in the necrotic center. This model could help explain why *Sclerotinia*-induced increased NPQ varies dependent on region and infection stage.

### The dynamics of NPQ in *Sclerotinia*-infected leaves

**Fig 2:**
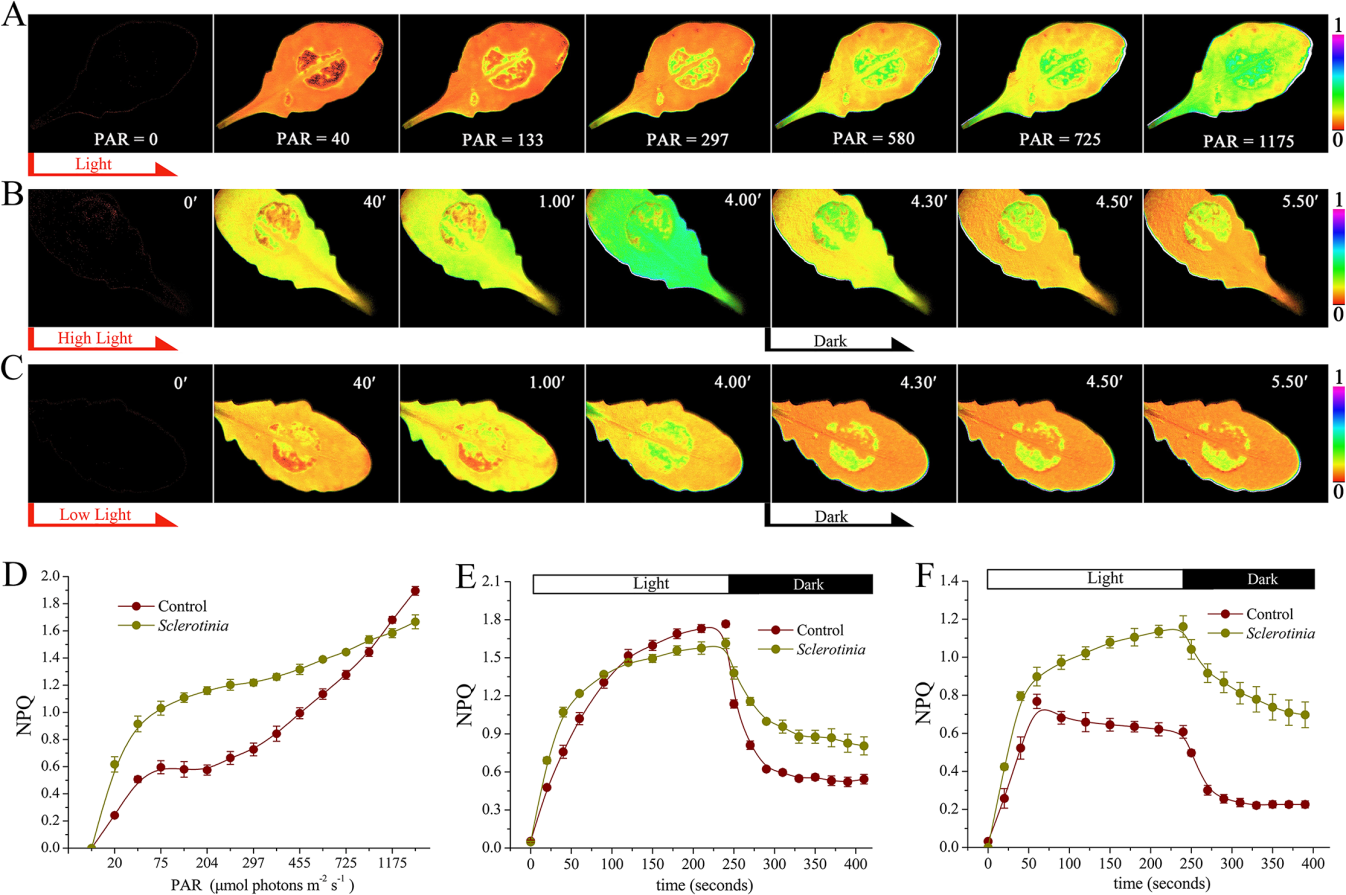
Dynamics of NPQ in *Sclerotinia*-infected leaves. (A-C) Chlorophyll fluorescence imaging showing changes of NPQ in *Sclerotinia*-inoculated leaves under (A) different intensity of actinic light, (B) high light (725 μmol photons m^-2^ s^-1^), and (C) low light (133 μmol photons m^-2^ s^-1^). PAR, photosynthetically active radiation (μmol photons m^-2^ s^-1^). Light was switched off at 4 min (black arrow). False colour code is depicted at the right of the image. (D) Kinetics of NPQ in *Sclerotinia*-infected leaves under different light intensity. (E, F) Induction and relaxation kinetics of NPQ in *Sclerotinia*-infected zone under high light (E) and low light (F). Each curve represents the average of three replicates ± _SE_.

We next analyzed the kinetic characteristics of NPQ in the *Sclerotinia*-infected leading edge area. First, different intensities of actinic light (levels of photosynthetically active radiation = PAR ranging from 0 to 1465 μmol photons m^-2^ s^-1^) were used to investigate dynamic changes in NPQ. As light intensity increased, a ring of enhanced NPQ was detectable surrounding the inoculated zone, and then expanded to the entire infected area (Fig 2A and S1 Movie). However, at light intensities exceeding 1175 μmol photons m^-2^ s^-1^, NPQ increased more in un-inoculated regions (Fig 2A and 2D). To analyze formation and relaxation of NPQ under excess light, a light intensity of 725 μmol photons m^-2^ s^-1^ was selected. In the *Sclerotinia*-infected zone, NPQ formed quickly in the first 60 seconds. In contrast, longer illumination led to greater NPQ generation in the un-inoculated control regions and with increased maximum amplitude (Fig 2B, 2E and S2 Movie). Interestingly, when the light switched off, NPQ relaxed more slowly in the infected area (Fig 2E).

Because NPQ has a dedicated function in protecting the photosynthetic apparatus against photodamage under excess light, we wanted to ascertain the impact of *Sclerotinia* infection on NPQ changes at the low light intensity of 133 μmol photons m^-2^ s^-1^, which was close to the natural radiation in our greenhouse. Surprisingly, even at this low light intensity, *Sclerotinia* infection still rapidly induced NPQ formation (Fig 2C, 2F and S3 Movie). More importantly, the maximal amplitude was approximately 2-fold of that found in un-inoculated regions (Fig 2F). Additionally, differences of NPQ relaxation kinetic between *Sclerotinia*-infected leaves and the control were still observed (Fig 2F). These results indicate that NPQ, usually seen under excess light conditions, was triggered by *Sclerotinia* invasion even at low light during early pathogenesis. To explore these effects induced by *Sclerotinia* under close to natural growth conditions, the low PAR of 133 μmol photons m^-2^ s^-1^ was used in the following experiments unless otherwise mentioned.

### The increase of NPQ in *Sclerotinia*-infected area is attributed to the decreased ambient pH

**Fig 3:**
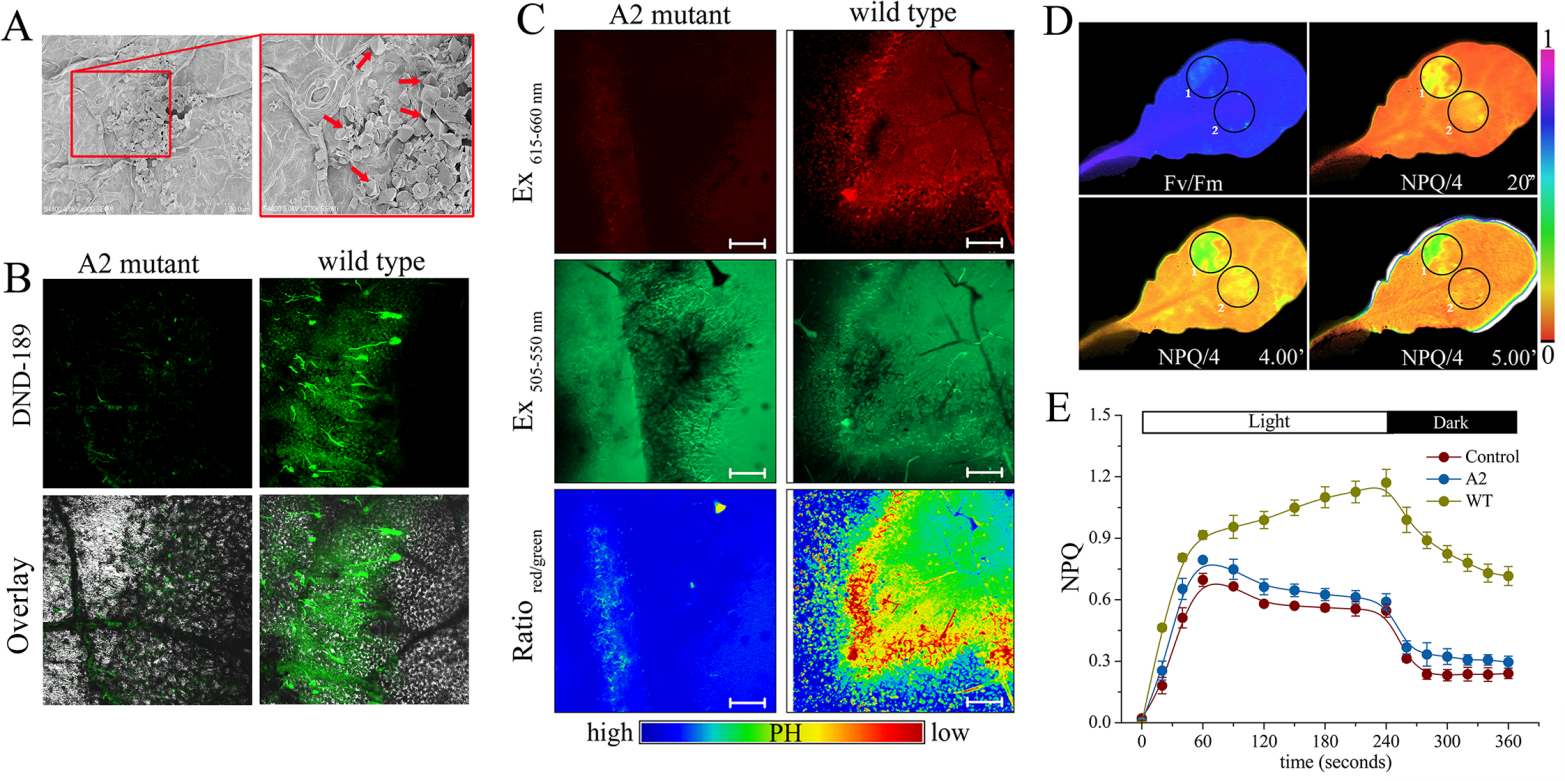
Role of *Sclerotinia*-secreted oxalate in the NPQ increase. (A) Scanning electron micrographs showing calcium oxalate crystal formed at the *Sclerotinia*-inoculated site. Red arrows indicate calcium oxalate crystal. (B, C) Confocal images showing positional pH changes in tissues infected with wild-type *Sclerotinia* or the oxalate-deficient A2 mutant. Tissue acidification was determined by lysosensor green DND-189 (B) and acridine orange (C), respectively. The fluorescence ratio of red to green emissions of acridine orange was displayed with pseudo-color. Bars = 50 μm. (D) False colour images showing Fv/Fm and NPQ/4 after challenge with wild-type *Sclerotinia* (Circle 1) and A2 mutant (Circle 2). (E) Dynamics of NPQ in A2 mutant and wild-type (WT) *Sclerotinia*-infected regions. Values represent average of three replicates ± _SE_.

Oxalate is an essential pathogenicity factor for *Sclerotinia* [52,57]. Inoculation with *Sclerotinia* induced calcium oxalate crystal accumulation in the infection sites (Fig 3A). It is controversial whether or not the dibasic acid oxalate aids in fungal invasion due to direct acidity effects. To explore whether the increased NPQ in *Sclerotinia*-infected zone was related to positional pH changes, two pH-sensitive fluorescent dyes, lysosensor green DND-189 and acridine orange, were used. The infected plant tissue exhibited a higher level of DND-189 fluorescence upon invasion with wild-type *Sclerotinia* compared to the oxalate-deficient A2 mutant (Fig 3B). Acridine orange has an emission maximum of 655 nm (red) in an acidic environment and of 530 nm (green) in a neutral environment [65]. Tissue acidification was determined by measuring the ratio of red-to-green emissions. Results obtained via confocal microscopy show that wild-type *Sclerotinia* infection induced a higher level of fluorescence emissions of acridine orange in the red channels (615 to 660 nm) compared to the A2 mutant. At the periphery of wild-type *Sclerotinia*-infected sites, the ratio of red-to-green emissions greatly increased (Fig 3C), indicating a decrease in ambient pH. We then evaluated whether loss of oxalate would affect the *Sclerotinia*-induced NPQ increase. One leaf was co-infected with wild-type *Sclerotinia* (Circle 1) and the oxalate-deficient A2 mutant (Circle 2). Chlorophyll fluorescence imaging revealed that the A2 mutant does not significantly stimulate NPQ as done by wild-type *Sclerotinia* (Fig 3D). The kinetics of NPQ formation and relaxation are similar in A2-inoculated leaves and controls (Fig 3E). These results suggest that acidification of the plant tissue by *Sclerotinia*-secreted oxalate might account for the abnormal increase in NPQ.

To further investigate the impact of ambient pH changes on NPQ kinetics, leaves were infiltrated with KOX (potassium oxalate) buffered to different pH values. A rapid increase in NPQ associated with a lower rate of NPQ relaxation was observed in leaves infiltrated with KOX at pH 3.0, versus pH 7.0 (S2A Fig and S4 Movie). These observations confirm that the decrease in ambient pH is responsible for the NPQ increase. Furthermore, inhibition of electron transport with 3-(3,4-dichlorophenyl)-1,1-dimethylurea (DCMU) abolished the enhanced NPQ seen in *Sclerotinia*-inoculated leaves (S2B Fig), indicating that the *Sclerotinia*-induced NPQ increase requires photosynthetic electron transport that is presumably coupled with translocation of H^+^ from stroma to lumen to generate a trans-thylakoid proton gradient (ΔpH). In contrast, in the *Sclerotinia*-inoculated region, dissipation of the pH gradient using the uncoupler nigericin did not fully abolish the NPQ increase like in the control (S2C Fig). We then measured ATP synthase activity to indirectly reflect the changes of proton motive force. Treatment with KOX at pH 3.0 significantly attenuated ATP synthase activity, as represented by decreased inorganic phosphate (Pi) at 630 nm (S2D Fig). A down-regulated ATP synthase activity was also confirmed by a bioluminescent luciferase assay detecting ATP generation (S3 Fig). Taken together, these results suggest that the decreased ambient pH caused by *Sclerotinia* infection might down-regulate ATP synthase activity by limiting proton flux into the stroma and enhancing thylakoid lumen acidification under illumination, thus resulting in increased NPQ.

### The components of zeaxanthin-related NPQ in *Sclerotinia*-infected leaves

**Fig 4:**
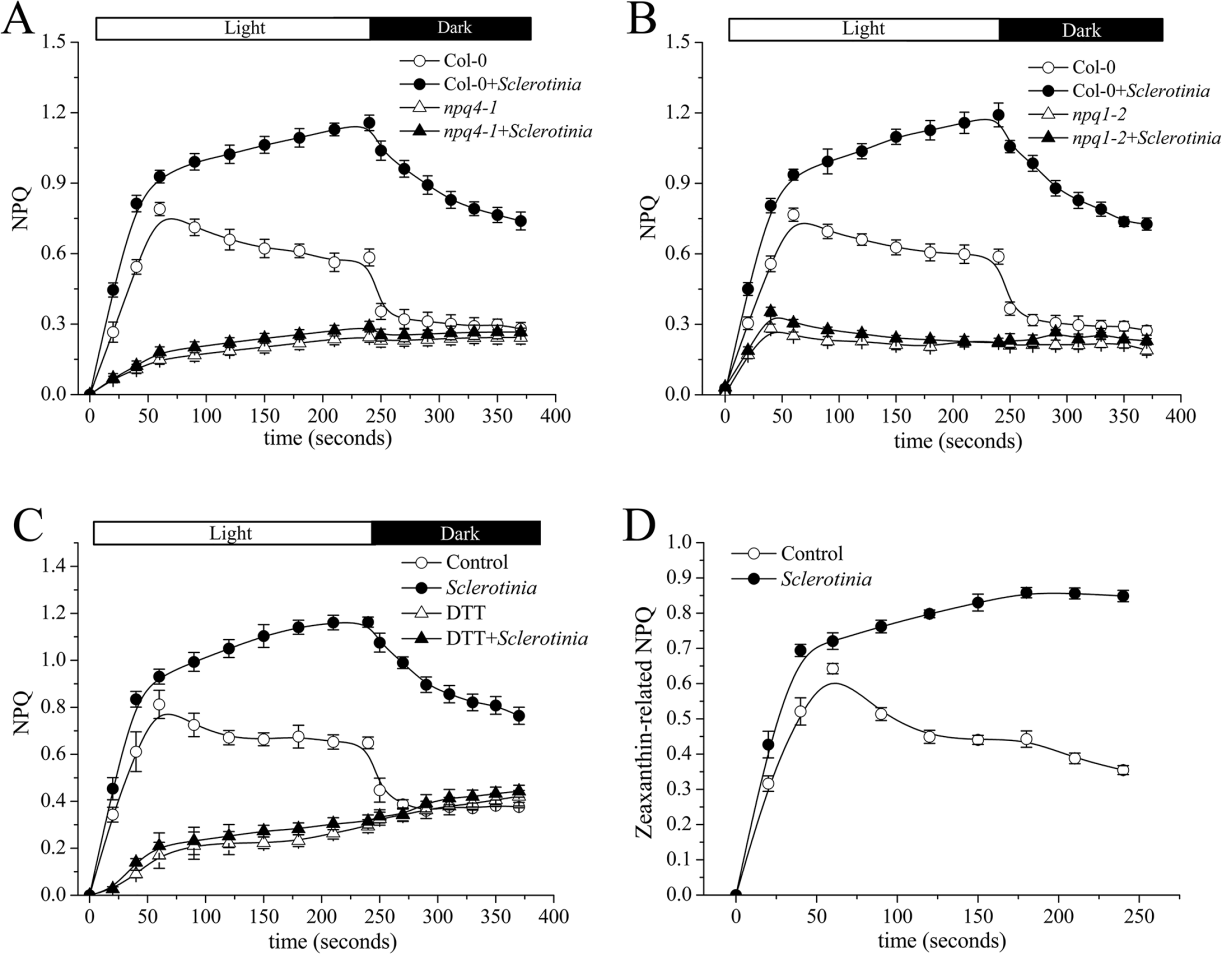
Effect of *Sclerotinia* invasion on the xanthophyll cycle. (A, B) Impact of mutations in *PsbS* (A) or *vde* (B) on the dynamics of NPQ induced by *Sclerotinia*. Kinetics of NPQ were recorded in *Sclerotinia*-infected *npq4-1* (*PsbS*) and *npq1-2*(*vde*) mutants, respectively. (C) The inhibitory effects of DTT on the kinetics of NPQ induced by *Sclerotinia*. NPQ was measured in dark-adapted Col-0 leaves after vacuum pre-infiltration with DTT (10 μM). (D) The component of zeaxanthin-related NPQ at *Sclerotinia*-infected regions. The amplitude of zeaxanthin-related NPQ was expressed as total NPQ minus NPQ+DTT. Data shown are the average of three replicates ± _SE_.

Since lumen acidification is sensed by the PsbS protein during NPQ generation [19], we next measured NPQ in a *Sclerotinia*-infected *PsbS* mutant (*npq4-1*). As expected, defective PsbS function greatly attenuated the abnormal increase in NPQ induced by *Sclerotinia* (Fig 4A). NPQ was induced slowly to a total extent of only 0.3, and most of this NPQ failed to relax during the subsequent period in the dark, consistent with the known NPQ defect of this mutant [18].

In addition to PsbS protein, other factors, such as the violaxanthin de-epoxidase (VDE), are required for full activation of NPQ [26]. A mutant deficient in VDE, *npq1-2*, is compared with Col-0 wild-type after inoculation with *Sclerotinia* in Fig 4B. In the *npq1-2* plant, *Sclerotinia* incubation did not induce increased NPQ, and the kinetic features consisted of a smaller increase and a slower second phase. After infiltration with dithiothreitol (DTT), a known inhibitor of VDE [16], NPQ formation was strongly inhibited in *Sclerotinia*-inoculated leaves (Fig 4C). Because VDE converts the carotenoid violaxanthin into zeaxanthin to participate in NPQ, we explored whether NPQ formation as associated with zeaxanthin level. Notably, *Sclerotinia* infection induced a high level of zeaxanthin-related NPQ (total NPQ kinetics minus NPQ kinetics + DTT [16,20]) (Fig 4D). Moreover, HPLC analysis showed that zeaxanthin increased 2.6-fold in *Sclerotinia*-infected Col-0 leaves compared to un-inoculated control leaves, with a corresponding decrease in violaxanthin content (Table 1). Conversely, presumably due to the loss of VDE, *Sclerotinia* infection did not promote greater zeaxanthin accumulation in the *npq1-2* mutant. Moreover, associated with the conversion of most of the violaxanthin to zeaxanthin, one of violaxanthin’s catabolites, neoxanthin, also decreased in *Sclerotinia*-infected leaves (Table 1). Collectively, these results indicate that the *Sclerotinia*-induced NPQ increase was closely correlated with VDE-catalyzed zeaxanthin generation.

**Table 1:** Pigment composition of *Sclerotinia*-infected plants.

	Vio	Ant	Zea	Neo	Lut	Chl *a/b*
Col-0	3.1 ± 0.3	0.4 ± 0.1	0.8 ± 0.2	4.0 ± 0.2	14.1 ± 1.0	3.10 ± 0.12
Col-0+*Sclerotinia*	0.9 ± 0.1	1.0± 0.2	2.2 ± 0.3	1.6 ± 0.6	13.6 ± 1.2	2.95 ± 0.06
*npq1-2*	3.2 ± 0.4	ND	ND	3.7 ± 0.4	14.0 ± 1.1	3.03 ± 0.10
*npq1-2*+*Sclerotinia*	2.8 ± 0.5	ND	ND	3.5 ± 0.1	13.5 ± 0.5	2.93 ± 0.07

Four-week-old *Arabidopsis* leaves were inoculated with *Sclerotinia* for 3 h and kept under a light intensity of 130 μmol m^-2^ s^-1^. Xanthophylls were subjected to HPLC analysis after extraction with pre-cooled acetone. Pigment content was normalized to 100 Chl *a*+*b* molecules, except for Chl *a*/*b*. Abbreviations: Vio, violaxanthin; Ant, antheraxanthin; Zea, zeaxanthin; Neo, neoxanthin; Lut, lutein; ND, not detectable. Data shown are means ± _SE_ (n = 3).

### The decrease in the xanthophyll precursors antagonizes ABA biosynthesis and the subsequent resistance to *Sclerotinia*

**Fig 5:**
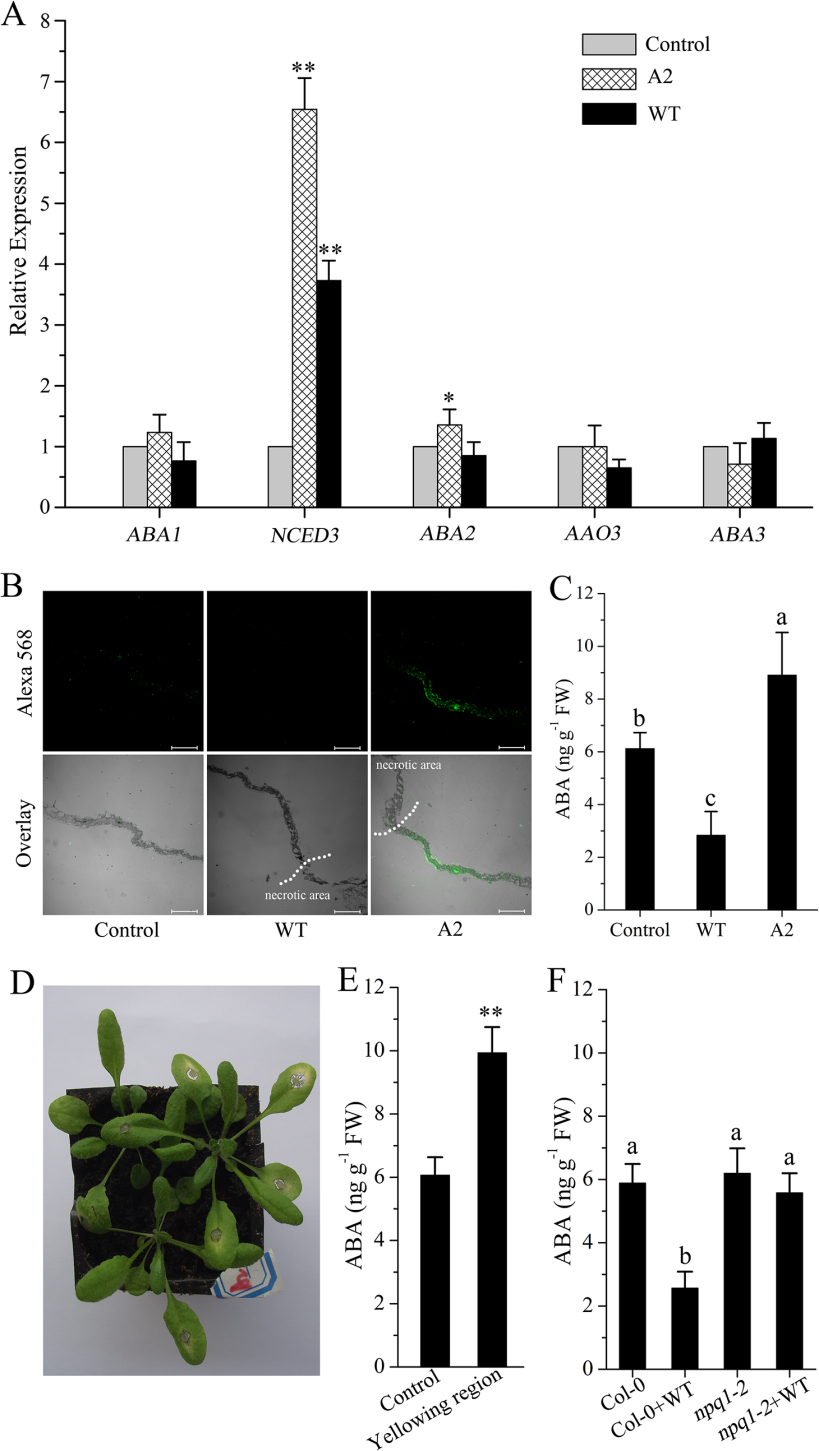
Impact of *Sclerotinia* invasion on ABA biosynthesis. (A) Effect of *Sclerotinia* invasion on the expression of ABA biosynthesis genes. The expression of target genes was determined by qPCR. (B) Immuno-cytochemical localization of ABA in longitudinal sections of *Arabidopsis* leaves. Bars = 0.2 mm. (C) Measurement of ABA levels in *Sclerotinia*-inoculated leaf regions with an ELISA Kit. (D) A2 mutant invasion caused leaf yellowing surrounding the necrotic lesions. Images were captured at 24 h after inoculation with A2 mutant. (E) Quantitative analysis of the content of ABA in the yellowing regions. (F) Mutation of VDE alleviated the decrease in ABA levels in *Sclerotinia*-infected *npq1-2* leaves. WT, wild-type *Sclerotinia*; A2, oxalate-deficient A2 mutant. (**), Student’s *t*-test significant at *P*< 0.01; (*), *P*< 0.05. Different letters indicate statistically significant differences (Duncan’s multiple range tests; *P*< 0.05). Data are average of three replicates± _SE_.

Because xanthophyll precursors (i.e. violaxanthin, neoxanthin) play a key role in ABA biosynthesis [37,38], we explored whether changes in these precursors may affect ABA metabolism. qPCR results showed that expression of ABA biosynthesis genes, except for *NCED3* (9-cis-epoxycarotenoid dioxygenases3), was not significantly affected after challenge with wild-type *Sclerotinia* or A2 mutant (Fig 5A). However, ABA immunoassays revealed that leaves incubated with the A2 mutant had elevated levels of ABA (Fig 5B), on average approximately 37% higher than controls (Fig 5C). In contrast, ABA levels in leaves inoculated with wild-type *Sclerotinia* were approximately 53% lower than those of control leaves (Fig 5C). In such circumstances, the expression of *NCED3* may be increased in an attempt to compensate for limitations in ABA biosynthesis. It is worth mentioning that A2 mutant invasion always resulted in tissue yellowing surrounding the necrotic area (Fig 5D). Statistical analysis showed that the yellowing region accumulated a high level of ABA (Fig 5E). We next analyzed the impact of defective of VDE on ABA synthesis. After growth for four weeks in the greenhouse, the *npq1-2* mutant showed similar ABA levels as Col-0. However, upon wild-type *Sclerotinia* infection, the mutation of VDE enzyme significantly suppressed the ABA levels decrease in the *npq1-2* mutant (Fig 5F). These results suggest that *Sclerotinia* infection could regulate the VDE activity to modify ABA levels.

**Fig 6:**
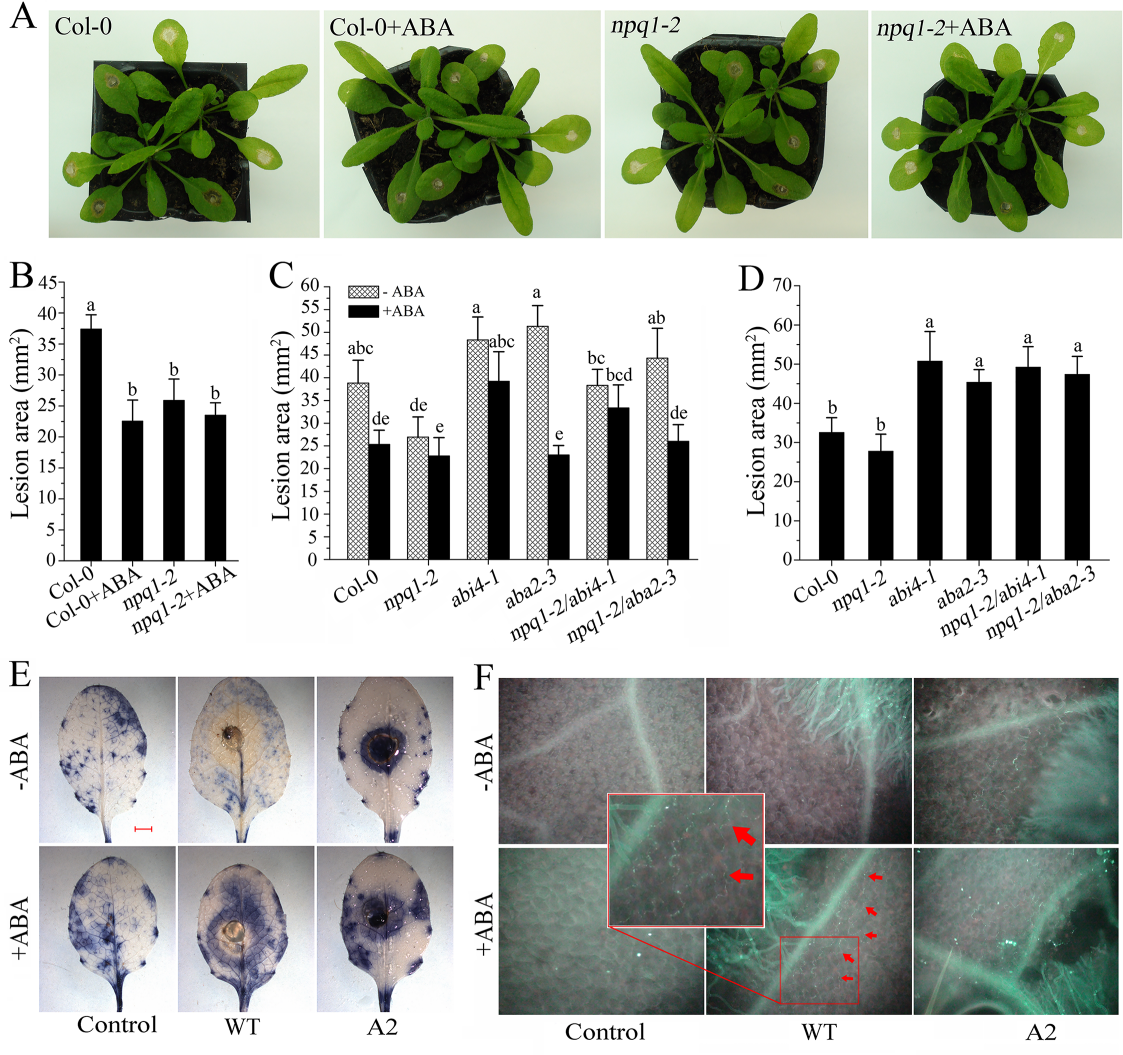
Efficiency of ABA in inducing plant resistance against *Sclerotinia*. (A, B) Effect of ABA on disease symptoms (A) and lesion areas (B) in Col-0 and *npq1-2* plants after inoculation with wild-type *Sclerotinia*. Leaves were incubated with *Sclerotinia* for 24 h in the presence or absence of 100 μM ABA. (C) Defects in ABA signaling increased susceptibility of *npq1-2*/*abi4-1* and *npq1-2*/*aba2-3* relative to *npp1-2* upon wild-type *Sclerotinia* infection. Lesion areas were measured at 24 h after inoculation with wild-type *Sclerotinia*. (D) Defects in ABA signaling increased susceptibility of *npq1-2/abi4-1* and *npq1-2/aba2-3* plants to oxalate-deficient A2 mutant. Lesion areas caused by the A2 mutant were measured at 48 h. Values represent means ± _SE_ of at least 10 lesions. Different letters indicate statistically significant differences (Duncan’s multiple range tests; *P* < 0.05). (E, F) Pretreatment with ABA induced O_2_^-^ formation (E) and callose deposition (F) at the advancing edge of necrotic areas. O_2_^-^ was stained with NBT, forming blue formazan. Red arrows indicate callose deposition. Scale bar = 2 mm. Images shown are representative.

We then evaluated the efficiency of ABA in plant resistance to *Sclerotinia*. Upon *Sclerotinia* infection, the average necrotic lesions in leaves pretreated with ABA were significantly smaller than in water-pretreated plants. Likewise, the infected *npq1-2* mutant showed a significant reduction in lesion area compared with Col-0, presumably due to the loss of VDE activity and associated maintenance of violaxanthin and/or neoxanthin levels and ABA synthesis (Fig 6A and 6B). However, due to defects in ABA sensing or ABA biosynthesis, the double mutants *npq1-2/abi4-1* and *npq1-2/aba2-3* were more susceptible to *Sclerotinia* than *npq1-2* plants. Interestingly, treatment with ABA reverted the phenotype of the *npq1-2/aba2-3* mutant but not the *npq1-2/abi4-1* mutant (Fig 6C). This result is consistent with previous reports [60], suggesting that ABI4 is involved in the downstream signaling of ABA in plant resistance to *Sclerotinia*. Mutation of either the *abi4* or the *aba2* gene significantly increased the infectious ability of the A2 mutant in *npq1-2/abi4-1* or *npq1-2/aba2-3* plants (Fig 6D). Together, these results clearly indicate that *Sclerotinia* could manipulate the xanthophyll cycle and interfere with ABA biosynthesis and signaling to suppress host defense.

To gain further insight into the nature of ABA-induced resistance against *Sclerotinia*, we assessed changes in defense responses, including O_2_^-^ formation and callose deposition. As shown in Fig 6E, O_2_^-^ was detectable surrounding the A2 mutant-infected zone that were either sprayed or not with ABA. However, in the wild-type *Sclerotinia*-inoculated leaves, only pretreatment with ABA was able to induce a ring of O_2_^-^ accumulation at the periphery of the infection site (Figs 6E and S4A). Upon A2 mutant incubation, callose deposition was also observed in the leading edge of the necrotic regions. Similar results were obtained in the vicinity of wild-type *Sclerotinia*-induced necrotic lesions after ABA treatment (Figs 6F and S4B). Since the rate of oxalate diffusion in leaf tissue is correlated with plant susceptibility to *Sclerotinia* [66], we presumed that ABA-induced local reinforcement of the cell wall by forming callose might be an effective physical barrier to prevent the spread of oxalate and limit mycelial growth.

## Discussion

### The impact of NPQ on plant disease resistance

Light acts as an initial signal that activates the special photoreceptors (e.g., phytochromes, cryptochromes and phototropins) involved in different types of plant-pathogen interactions [6–7, 67]. In fact, the mechanisms that directly control photosynthetic light reactions also mediate important functions in plant response to pathogens [6, 68]. Recently, it has been reported that the light-induced reduction of the plastoquinone pool may trigger induction of defense-associated genes and the hypersensitive reaction [2, 69]. To manipulate such redox signals, pathogens may modulate the activation state of photoprotective mechanisms like changing the extent of NPQ, as an effective way to alter the redox status of the plastoquinone pool and generation of ROS from the photosynthetic electron transfer chain [68]. However, the impact on NPQ by different pathogens seems different, either increasing or decreasing NPQ [34–35]. At present, there is still no clear recognition of these pathogens’ role in manipulating NPQ. The measured NPQ under strong light is just as an indicator for early detection of pathogens in leaves [35,64]. Here, our results demonstrate for the first time that *Sclerotinia* infection induces a localized increase in NPQ in early pathogenesis under the natural and rather low growth light intensity (Figs 1 and 2). While there are a few reports of pathogens inducing NPQ under such low light intensity, not much is known about an involvement of this effect in the pathogenic process. The present data, however, revealed that an increased NPQ under such low light conditions caused changes in downstream cellular events, such as ABA biosynthesis and ROS generation, which weaken host defense responses during the natural pathogenic processes of *Sclerotinia* (Figs 5 and 6). Thus, the localized increase in NPQ in the early stage of infection cannot be regarded as a consequence of the metabolic perturbation of the host cell. Rather, this process is likely proactive in aiding pathogenic success. A more systematic study is required to validate this assumption.

### Tissue acidification and zeaxanthin conversion are necessary for *Sclerotinia*-induced NPQ increase

Many pathogens are able to actively increase or decrease the surrounding pH at the infection site through alkalization or acidification [48–49]. For *Sclerotinia*, a low environmental pH may aid pathogenicity by affecting numerous pH-regulated genes and cell-wall-degrading enzymes [49–53]. As an early pathogenic event, the *Sclerotinia*-induced increase of NPQ demonstrated here in host plant is also closely related to a low ambient pH (Fig 3). Interestingly, incubation with another oxalate-secreting fungus, *Botrytis cinerea*, also induced an NPQ increase, although to a much smaller extent than that in *Sclerotinia*-inoculated regions (S5 Fig). Such differences might result from *B*. *cinerea* secreting lower amounts of oxalate than *Sclerotinia* [70]. However, for pathogens (e.g., *Pseudomonas syringae*) which cannot secrete acidic or alkaline factors, it is still unclear how they affect NPQ during their pathogenic progress [31–32]. NPQ increases or decreases in response to the level of light utilization in photosynthesis, and any impact of a pathogen on sugar export or sugar consumption is expected to result in changes in NPQ [12, 71]. In addition, NPQ is directly controlled by the trans-thylakoid proton gradient by at least two processes requiring a low lumenal pH, i.e., (i) the induction of VDE activity (and resulting conversion of violaxanthin to zeaxanthin) and (ii) the protonation of the PsbS protein leading to the engagement of zeaxanthin in the actual dissipation process [12,16–17,27]. It is noteworthy that the decrease in lumenal pH that induces NPQ does not have to be generated by photosynthetic electron transport [26,45,47]. Using isolated thylakoids, NPQ can be induced in darkness by simply lowering buffer pH [46]. While inhibition of linear electron flow with DCMU abolished the NPQ features induced by *Sclerotinia*, proton gradient collapse by treatment with the uncoupler nigericin did not fully abolish the *Sclerotinia*-induced NPQ increase (S2B and S2C Fig). Our data suggest that early *Sclerotinia* infection down-regulates ATP synthase activity and thereby leads to a decreased lumen pH and increased NPQ.

Furthermore, infected areas showed NPQ dynamics similar to those of *aba1-3* (S6 Fig), a mutant deficient in the ZEP enzyme of the xanthophyll cycle that is associated with constitutive accumulation of zeaxanthin [20,72]. Consistent with the features of the latter mutant, our HPLC analyses of Col-0 leaves incubated with *Sclerotinia* revealed a significant increase in zeaxanthin content (Table 1). Accumulation of zeaxanthin is necessary to modulate the kinetics of NPQ, enhancing the rate of NPQ formation and retarding the rate of NPQ relaxation [72]. This effect can explain why inhibition of zeaxanthin formation abolished the *Sclerotinia*-induced NPQ increase in the *npq1-2* mutant or wild-type leaves treated with DTT (Fig 5B and 5C).

### *Sclerotinia*-induced modulation of the xanthophyll cycle curbs ABA biosynthesis

Biosynthesis of ABA begins inside the chloroplast and is limited by xanthoxin synthesized from violaxanthin [36–37,39]. In strong light, a low lumen pH activates VDE-catalyzed deepoxidation of violaxanthin to zeaxanthin [17,26]. Restraint of VDE activity results in violaxanthin accumulation and promotes ABA synthesis [4]. In the *vtc1* mutant with a reduced level of the VDE substrate ascorbate, ABA content increased by 60% compared to that of wild-type [43]. Consequently, a reduction in ABA closely matched the decrease in the amounts of violaxanthin plus neoxanthin after infection with *Sclerotinia* (Table 1 and Fig 5). However, qPCR analysis showed that, except for *NCED3*, the expression of ABA biosynthesis genes was not significantly affected by wild-type *Sclerotinia* infection (Fig 5A). This suggests that, in the early stage of infection, regulation of ABA biosynthesis occurs primarily at the substrate level (violaxanthin) rather than at the transcriptional level. Since NCED3 is the key enzyme in the ABA biosynthesis pathway [39,73], the increase in *NCED3* expression might result from the demand for ABA in the infected tissues. *Sclerotinia* infection generates the acidic environment that increases activation of VDE, promoting conversion of violaxanthin to zeaxanthin. The decrease in the ABA precursor violaxanthin may be the main reason for the reduced ABA levels in *Sclerotinia*-infected leaf discs. Taken together, our results suggest that modulation of the xanthophyll cycle provides a mechanism to adjust production of ABA for signaling purposes. To further evaluate this conclusion, it would be interesting to determine the interplay of lumen pH and/or sugar accumulation (and resulting NPQ changes) with ABA-mediated defense signaling in many other pathogens that can up-regulate or down-regulate NPQ in their pathogenic processes [32,34–35]. Additionally, it is worth mentioning that *Sclerotinia* infection caused stomatal pores to be more widely open within and around necrotic lesions after dark adaptation [59–60]. Because stomatal movement is tightly regulated by ABA-mediated signaling (such as ROS generation, Ca^2+^ permeable cation channels regulation) [74], decreased ABA levels might offer an explanation for the inhibitory action of *Sclerotinia* on stomatal closing.

### Tissue defense responses induced by ABA participate in plant resistance to *Sclerotinia*

Although ABA’s role in influencing the outcome of plant-pathogen interactions is controversial, functional genetic studies have provided evidence for a positive role of ABA in defense against *Sclerotinia* [60–63]. In agreement with this view, exogenously applied ABA significantly restricted development of necrotic lesions caused by *Sclerotinia*. Importantly, the *npq1-2* mutant showed more resistance to *Sclerotinia* compared with Col-0 plants (Fig 6A and 6B). It is likely that *Sclerotinia* cannot manipulate the xanthophyll cycle in *npq1-2* plants due to their deficiency in the VDE enzyme, thus leading to unchanged ABA levels. This hypothesis was confirmed by measuring ABA levels in *npq1-2* (Fig 5F). However, the defect in either ABA sensing or ABA biosynthesis weakened the resistance effect in *npq1-2/abi4-1* or *npq1-2/aba2-3* plants upon *Sclerotinia* infection (Fig 6C). The oxalate-deficient A2 mutant is less pathogenic than wild-type fungus [52]. Compared to wild-type *Sclerotinia*, the A2 mutant did not show significantly reduced susceptibility in *npq1-2* (Fig 6D), which might result from the already higher levels of ABA induction in the plant response to A2 mutant infection (Fig 5B–5E). This could explain why the *abi4* or *aba2* mutations increased *npq1-2* plant susceptibility to the A2 mutant (Fig 6D). Curiously, although silencing the NPQ machinery with mutant or inhibitor delayed the progression of lesion expansion, both wild-type *Sclerotinia* and the A2 mutant were still capable of infecting living plant tissue. One reason for this might be the use of PDA agar, which always leads to aggressive growth of *Sclerotinia* and overwhelms the defense capacity of plant. Actually, disease caused by this devastating necrotrophic fungal has traditionally been difficult to control [53]. Even if situated under unfavorable conditions, *Sclerotinia* could survive with the aid of sclerotia. Therefore, one can imagine that if the fungus is not directly killed at the source, it can breakthrough an already established defense system. Manipulation of NPQ in the pathogenic process of *Sclerotinia* is an early event, which primes other invasion processes like suppression of oxidative burst. Thus, we assume that the actual role of the *Sclerotinia*-induced increasing NPQ may contribute to successful early infection establishment.

ROS function as important second messengers in ABA-mediated defense signaling [75]. Manipulation of ROS signals in the pathogenic process of *Sclerotinia* is particularly intriguing. *Sclerotinia*-secreted oxalate initially suppressed host oxidative burst, but later promoted ROS generation to achieve pathogenic success [57–58]. Undoubtedly, inhibition of early ROS signaling can contribute to restraining plant activation of defense responses and favor of *Sclerotinia* invasion. At present, the physiological and molecular regulation mechanism of the initial ROS inhibition is not very clear. Although the previous research reported that the inhibitory effects of oxalate on ROS are largely independent of its acidity, lowering the medium pH indeed led to a greater inhibition of oxidative burst [57]. Tissue acidification is sufficient for *Sclerotinia* inducing an increase in NPQ, which is known to minimize production of ^1^O_2_* in the PSII antenna [15,26]. Moreover, NPQ is also correlated with the activation of photosynthetic control, which limits electron transport through the cytochrome b6f complex and alleviates the formation of ROS in PSI [68]. In addition to the effect of thermal dissipation (leading to NPQ) in causing de-excitation of singlet-excited chlorophyll, and thereby decreasing ROS formation, there is also a direct effect of zeaxanthin in deactivating ROS and their effect on biological membranes [33]. The *Sclerotinia*-induced increase in NPQ and in zeaxanthin accumulation was indeed paralleled by suppression of O_2_^-^ generation (Figs 1A–1E and 6E). In the early infection stage, there seems to be a correlation between the NPQ increase (and zeaxanthin accumulation) and oxidative burst inhibition. The *Sclerotinia*-induced increase of NPQ (and zeaxanthin accumulation) under low light should also be expected to attenuate ROS generated in the photosynthetic light reactions. In fact, there is a background level of triplet chlorophyll formation and potential singlet-oxygen formation even under low light level since the fraction of absorbed light converted to photosynthetic electron transport does not exceed about 85% [76]. It is worth noting that exogenous application of ABA reversed the decrease of O_2_^-^ (Fig 6E), possibly via activation of other cellular ROS-generating systems like NADPH oxidases. The latter mutations were showed susceptible to *Sclerotinia* in previous report [62]. Further studies are required to test this hypothesis.

**Fig 7:**
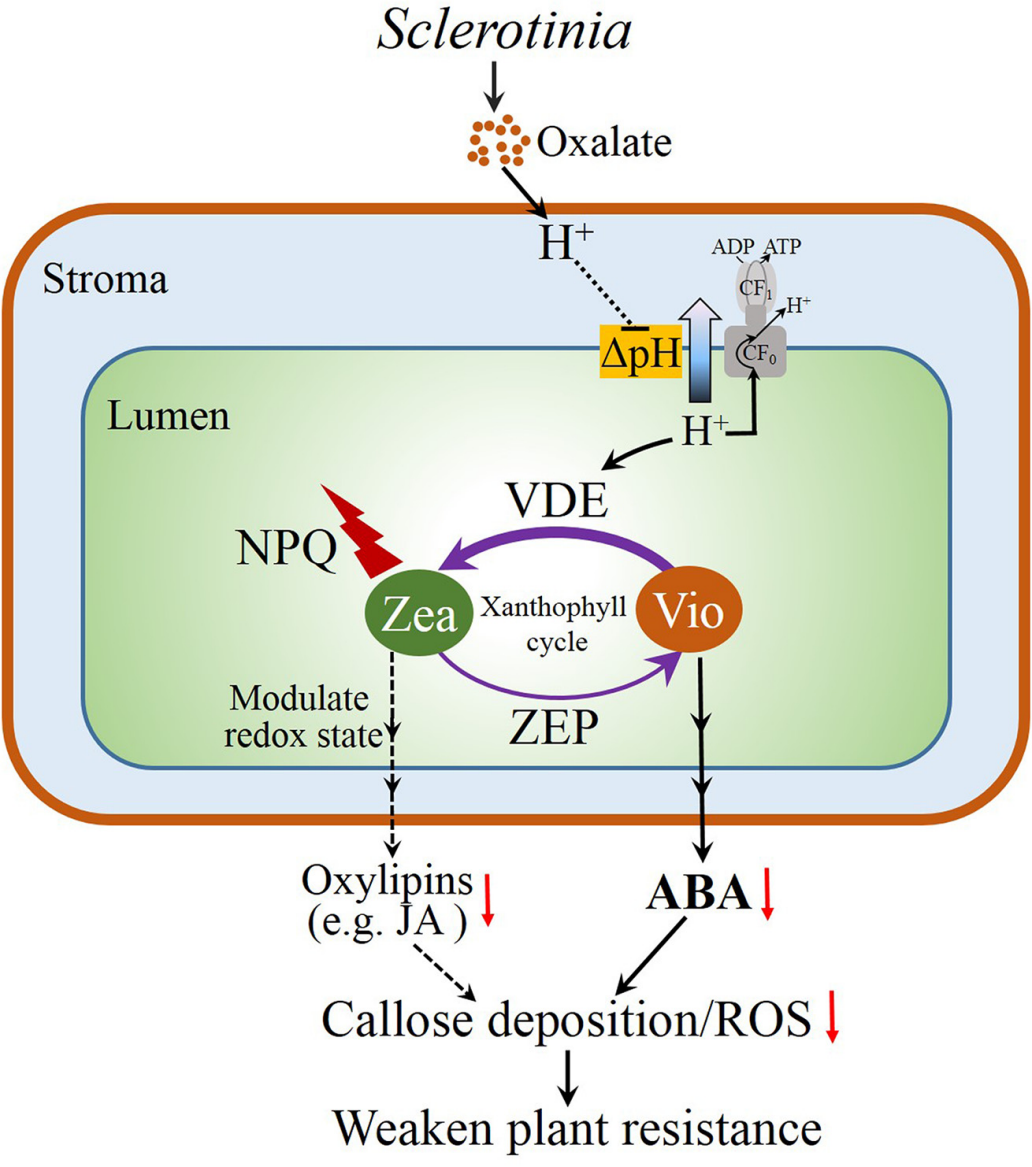
Proposed model for the interplay of the xanthophyll cycle and plant resistance to *Sclerotinia*. *Sclerotinia* secretes oxalate to acidify the infected tissues, which results in increased lumen acidification. Subsequently, the low lumen pH activates NPQ by protonating PsbS protein and VDE. The latter convert violaxanthin (V) to zeaxanthin (Z) via the intermediate antheraxanthin (A) in the xanthophyll cycle. Elevated NPQ and zeaxanthin accumulation have the potential to affect chloroplast redox singling pathways to lower oxidation-derived oxylipins (like jasmonic acid, JA) formation [10–11,77]. On the other hand, the decrease in precursor violaxanthin limits ABA biosynthesis. These aspects, in turn, affects tissue defense responses like ROS induction and callose deposition, which increases plant susceptibility to *Sclerotinia*.

In summary, our investigation provides evidence about an interplay of the xanthophyll cycle and plant resistance against the necrotrophic pathogen *Sclerotinia*. The possible correlations are summarized in the model presented in Fig 7. Initially, *Sclerotinia* secretes oxalate to acidify the infected tissues, which down-regulates ATP synthase activity and increases NPQ by protonating PsbS protein and activating the VDE enzyme. The latter goes on to convert violaxanthin to zeaxanthin via the intermediate antheraxanthin in the xanthophyll cycle. The decrease in precursor violaxanthin limits ABA biosynthesis and, in turn, affects tissue defense responses including ROS induction and callose deposition, which increase plant susceptibility to *Sclerotinia*. Additionally, the excellent works of Demmig-Adams & Adams group indicate that elevated NPQ and zeaxanthin accumulation have the potential to affect chloroplast redox status to lower oxidation-derived oxylipins (like jasmonic acid) formation [10–11,77], which may weaken callose formation and occlusion of sugar-loading complexes, presumably to facilitate pathogen spread via the phloem. In conclusion, the present study reveals a novel perspective on infection strategies of the necrotrophic fungal *Sclerotinia*, which provides a model of how photoprotective processes and metabolites are integrated into the plant defense network and thereby contributes to a better understanding of early plant-*Sclerotinia* interactions at the infection sites.

## Materials and Methods

### Plant material and chemicals treatment

*Arabidopsis* Columbia-0 (Col-0), *abi4-1* (N8104), *aba2-3* (N3834), *npq1-2* (N3771) and *npq4-1* (N66021) were obtained from the European Arabidopsis Stock Centre. For the generation of crosses *npq1-*2/*abi4-1* and *npq1-*2/*aba2-3*, *abi4-1* and *aba2-3* mutants were directly crossed to *npq1-*2. F2 seeds were germinated on agar plates in the presence of 5 μM ABA. The seedlings with expanded cotyledons were screened via fluorescence video imaging (PAM-MINI, Walz, Germany). F3 lines were re-screened to identify true mutants, and F3 seedlings were used for experiments. Plants were cultivated in growth cabinets at 22°C with a 16-h photoperiod and a light intensity of 120 μmol photons m^-2^ s^-1^. The chemicals ABA, DTT and DCMU were purchased from Sigma-Aldrich; nigericin was obtained from J&K Scientific Ltd.. Detached leaves from 4-week-old plants were inoculated with *Sclerotinia* at 1 h after vacuum pre-infiltration with DCMU (8 μM), nigericin (50 μM) or DTT (10 μM), respectively. For ABA treatment, leaves were sprayed with 100 μM of cis, trans-ABA (dissolved in 0.1% (v/v) ethanol) at 24 h prior to *Sclerotinia* inoculation. Control leaves were sprayed with water containing 0.1% (v/v) ethanol.

### Lesion area measurement

Wild-type *Sclerotinia* and an oxalate-deficient mutant (A2) were cultivated on potato dextrose agar at 21°C for 3 days. Agar plugs (diameter 0.3 or 0.8 cm) containing the leading edge of growing mycelia were used to inoculate leaves. Infected plants were kept under saturating humidity conditions in a clear plastic box. Photos of necrotic phenotype were captured by a numeric camera (HDR-XR500E, Sony). Lesion size was quantified in at least 10 leaves with a Carl Zeiss system as described by [59], with photographs captured by a Carl Zeiss AxioCam MRc5 camera installed on a Zeiss inverted microscope. Lesion area was quantified with the measurement tool ‘outline spline’ in AxioVision Rel.4.5 software. A measured example is given in S7 Fig.

### Measurement of chlorophyll fluorescence parameters

Chlorophyll fluorescence parameters were measured with an Imaging-PAM Chlorophyll Fluorometer (PAM-MINI, Walz, Germany). The experimental procedures were as described previously [20, 32]. After inoculation with *Sclerotinia*, leaves were dark-adapted for 1 h prior to measurement. Parameters Fo (minimum fluorescence with PSII reaction centers fully open), Fm (maximum fluorescence after dark adaptation) and Fm’ (fluorescence level under actinic light-adapted state) were acquired by the ImagingWin software (ImagingWin v2.0m, Walz) (S8 Fig). A 0.8-s saturating pulse (4,000 μmol photons m^-2^ s^-1^) was applied to obtain Fm and Fm’. Fv/Fm and NPQ were automatically calculated by the ImagingWin software (Walz) using the formulas (Fm-Fo)/Fm and (Fm-Fm’)/Fm’, respectively. Actinic light of 725 μmol photons m^-2^ s^-1^ was selected as high light, and 133 μmol photons m^-2^ s^-1^ was used as low light. Images of the fluorescence parameters were displayed with a false color code, ranging from zero (black) to one (purple).

### Investigation of the infection process by scanning electron microscope

After inoculated with *Sclerotinia* for 1h or 12h, the leaves were immediately fixed in formalin-acetic acid-alcohol for 24 h [78]. Samples were then washed three times in distilled water. Sections were next dehydrated through an ethanol series (70%, 80%, 90%, 95% and 100%; 30 min at each step). Ethanol-dehydrated samples were processed with critical point drying followed by platinum coating. Coated samples were scanned with a cold field scanning electron microscope at an accelerating voltage of 3.0 kV (S-4800, Hitachi).

### Positional tissue acidification measurement

Positional pH was measured according to [65]. *Sclerotinia*-infected leaves were stained with LysoSensor Green DND-189 (2.5 μM) for 20 min or with acridine orange (50 μM) for 1.5 h. Stained leaves were washed twice with a washing buffer (10 mM KCl, 10 mM MES, pH 6.05). Fluorescence of DND-189 was acquired using a Zeiss LSM510 META system at excitation/ emission wavelengths of 458 nm/505 to 530 nm. Fluorescence emissions of acridine orange in red (615 to 660 nm) and green channels (505 to 550 nm) were obtained after excitation with 488 nm. Tissue acidification was represented by the ratio of the red-to-green emissions of acridine orange.

### Qualitative measurement of ATP synthase activity

ATP synthase activity was assessed by determining the decrease in the concentration of inorganic phosphate (Pi) according to [79]. In brief, 10 mM KOX at a pH of 7.0 or 3.0 was incubated with 0.1 mg Chl ml^-1^ chloroplasts suspension (110 mM sorbitol and 17 mM Hepes-KOH, pH 8.0). After adding ADP (4 mM) and Pi (50 μM), chloroplasts were illuminated with 130 μmol photons m^-2^ s^-1^ for the indicated time (0–5 min) and stopped by addition of 4% (w/v) cold trichloroacetic acid. Samples were then mixed with 0.65 M sulfuric acid and 8.5 mM ammonium molybdate, followed by measuring absorbance at 630 nm (PerkinElmer, Lambda35, UK).

### Pigment analysis

*Sclerotinia*-infected plants were kept under saturating humidity and a light intensity of 130 μmol m^-2^ s^-1^. Infected areas of leaves were obtained with a hole punch at 3 h after inoculation. Pigments were immediately extracted with pre-cooled acetone under dim light condition [80]. After filtering with a 0.2 μm filter, the extract was separated and quantified by HPLC with a Waters Spherisorb S5 ODS1 column (5.0 μm, 4.6 mm × 250 mm). The solvent system was according to Müller-Moulé et al. [81]. Solvent A (acetonitrile: methanol: Tris-HCl 0.1 M pH 8.0 [84: 2: 14]) was eluted with a linear gradient to 100% solvent B (methanol: ethyl acetate [68: 32]) within 15 min, followed by 5 min of solvent B. Relative contents were normalized to 100 chlorophyll *a*+*b* molecules.

### Quantitative real-time PCR (qRT-PCR) analysis

Total RNA was extracted from *Sclerotinia*-infected zone using RNAiso Plus (Takara, Dalian, China) according to the supplier’s recommendation. First-strand cDNA was synthesized with the SuperScript II First-Strand Synthesis System (Invitrogen). qRT-PCR was performed using the lightCycler (Roche) real-time PCR detection system. Primer sequences (S1 Table) of *ABA1* (At5g67030), *ABA2* (At1g52340), *ABA3* (At1g16540), *NCED3* (At3g14440) and *AAO3* (At2g27150) were used as described by [82]. Expression of target genes was normalized to *ACTIN2*.

### Measurement of ABA

ABA extraction was according to [83]. Briefly, *Sclerotinia*-infected leaf discs were collected and frozen in liquid nitrogen. After grinding with a pre-chilled mortar and pestle on ice, the powder was extracted overnight at 4°C in a cold extraction buffer (80% methanol and 2% glacial acetic acid). The mixture was then centrifuged at 2,000 g for 5 min. The supernatant was run through a Sep-Pak C18 Plus Short Cartridge (Waters Corp) to remove polar compounds. ABA content was measured by a plant ABA ELISA Kit (Jiancheng, Nanjing, China).

Tissue immuno-localization of ABA was according to [84]. *Sclerotinia*-infected leaves were fixed overnight with 3% (W/V) para-formaldehyde in 4% (W/V) 1-ethyl-3-(3-dimethylaminopropyl) carbodiimide containing 0.1% (V/V) Triton X-100. Tissue cleaning was performed before infiltrating with wax. Sections (12 μm) were obtained with a sliding microtome (CM1850, Leica, Germany). After dewaxing and blocking, sections were incubated with rabbit anti-ABA primary antibody (Agrisera, Vännäs, Sweden) overnight. The fluorescence of Alexa 568 conjugated anti-rabbit secondary antibody was viewed with a confocal microscope (Zeiss LSM510 META).

### Measurement of superoxide anion (O_2_^-^) generation and callose deposition

Accumulation of O_2_^-^ was monitored *in situ* with nitroblue tetrazolium (NBT) as described previously [85]. Images were photographed using a Zeiss inverted microscope with a Carl Zeiss AxioCam MRc5 camera. Callose deposition was stained with 0.01% (w/v) aniline-blue and observed using a fluorescent microscope [86].

## Supporting Information

S1 Fig Light micrographs showing the development of infection cushions. Leaves were stained with Coomassie Brilliant Blue R-250. The staining procedure was performed as described previously [87]. The images were captured with an Mshot CCD MS31.
(TIF)

S2 Fig Changes in thylakoid proton gradient upon *Sclerotinia* infection. (A) Different kinetics of NPQ induced by KOX at pH 7.0 and 3.0. Detached leaves were syringe-infiltrated with 10 mM KOX buffered to 7.0 or 3.0 with HCl. (B, C) Impact of DCMU (B) and nigericin (C) on the kinetics of NPQ induced by *Sclerotinia*. Detached leaves were vacuum-infiltrated with DCMU (8 μM) or nigericin (50 μM). After inoculation with *Sclerotinia*, these leaves were dark-adapted for 1 h prior to measurement of NPQ. (D) KOX at pH 3.0 partially inhibited ATP synthase activity. After adding 4 mM ADP and 50 μM Pi, chloroplast suspensions were illuminated with 130 μmol photons m^-2^ s^-1^ for the indicated time. ATP synthase activity was monitored by colorimetric determination of the decrease in Pi at 630 nm. Values are means ± _SE_ of three replicates.
(TIF)

S3 Fig Effect of low ambient pH on ATP synthase activity. (A) KOX at pH 3.0 reduced the ATP synthase activity. (B) The effect of pH without KOX on ATP synthase activity. The ATP synthase activity were qualitatively measured by detecting ATP generation with firefly luciferin-luciferase reaction. After illumination with light (130 μmol photons m^-2^ s^-1^) for the indicated time (0–3 min), the solution was re-adjusted to pH 7.8 and added D-luciferin (100 μM) and luciferase (100 μg/mL). The luminescence was recorded by an ICCD (576S-1, Princeton) over a 5 min period.
(TIF)

S4 Fig Effect of exogenous ABA on the tissue defense responses. Pretreatment of ABA induced O_2_^-^ formation (A) and callose deposition (B) around the developing necrotic lesions. Arrows indicate the leading edge of callose deposition. NBT staining were photographed by a SONY numeric camera (HDR-XR500E). The images of callose deposition were captured with an Mshot CCD MS31. Three repetitions were given in each treatment groups.
(TIF)

S5 Fig Effect of *B*. *cinerea* infection on the changes in NPQ kinetic. (A) Chlorophyll fluorescence image shows NPQ changes in *B*. *cinema*-infected *Arabidopsis* leaves. (B) Induction and relaxation kinetics of NPQ in *B*. *cinema*-infected zone. The light is switched off after 240 seconds. Each curve represents the average of three replicates ± _SE_.
(TIF)

S6 Fig Effect of zeaxanthin accumulation on the dynamics of NPQ. (A) Chlorophyll fluorescence image shows the difference of NPQ formation in *aba1-3* and Ler-0 leaves. (B) Induction and relaxation kinetics of NPQ in *aba1-3* and Ler-0 leaves. Actinic light of 725 μmol photons m^-2^ s^-1^ was selected to measure the dynamics of NPQ. The light is switched off after 200 seconds. Each curve represents the average of three replicates ± _SE_.
(TIF)

S7 Fig An example for the lesion area measurement. Photographs were initially captured using a Zeiss inverted microscope installed with a Carl Zeiss AxioCam MRc5 camera. Lesion area was quantified with the measurement tool ‘outline spline’ in AxioVision Rel.4.5 software. The unit of measurement is micrometer (μm). Bar = 2 mm.
(TIF)

S8 Fig The original fluorescence parameters in *Sclerotinia*-infected *Arabidopsis* leaf. (A) Chlorophyll fluorescence image shows NPQ changes after infection with *Sclerotinia*. (B) The original trace of current fluorescence yield (Ft) in *Sclerotinia*-infected leaf. Because Ft is not continuously stored in the Buffer-Memory, a direct screenshot from the ImagingWin software was given. To facilitate comparison, the control (circles 3 and 4) here was selected from the un-inoculated region. Fo, Fm and Fm’ were marked in the Ft trace. “AL on” refer to turning the continuous actinic light (133 μmol photons m^-2^ s^-1^) on. The changes of NPQ/4 were showed below with red lines.
(TIF)

S1 Table qPCR primers used for determining ABA biosynthesis genes. (DOCX)

S1 Movie Movie shows the kinetics of NPQ in *Sclerotinia*-infected leaves under different light intensity. PAR, photosynthetically active radiation (μmol photons m^-2^ s^-1^). Each picture was captured with 20-second intervals using an Imaging-PAM Chlorophyll Fluorometer. The pictures shown in the movie are representative.
(MOV)

S2 Movie Movie shows the dynamics of NPQ in *Sclerotinia*-infected leaves at excess light. 725 μmol photons m^-2^ s^-1^ was selected as excess light intensity. The light is switched off at 4 min. Images were captured at the indicated time point with an Imaging-PAM Chlorophyll Fluorometer. The pictures shown are representative.
(MOV)

S3 Movie Movie shows the dynamics of NPQ in *Sclerotinia*-infected leaves at low light. Light intensity at 133 μmol photons m^-2^ s^-1^ was selected as low light. The light is switched off at 4 min. Chlorophyll fluorescence images were captured at the indicated time point using an Imaging-PAM Chlorophyll Fluorometer. The pictures shown are representative.
(MOV)

S4 Movie Movie shows the dynamics of NPQ in leaves infiltrated with different pHs of KOX. KOX was adjusted to pH 7.0 and 3.0 with HCl. The light is switched off at 4 min. Images were captured at the indicated time point with an Imaging-PAM Chlorophyll Fluorometer. The pictures shown are representative.
(MOV)
