# Supplementary figures and images for: Manipulation of the Xanthophyll Cycle Increases Plant Susceptibility to Sclerotinia sclerotiorum
Source: PLoS Pathog. 2015 May 20;11(5):e1004878. doi: 10.1371/journal.ppat.1004878 (PMC4439079; doi:10.1371/journal.ppat.1004878)

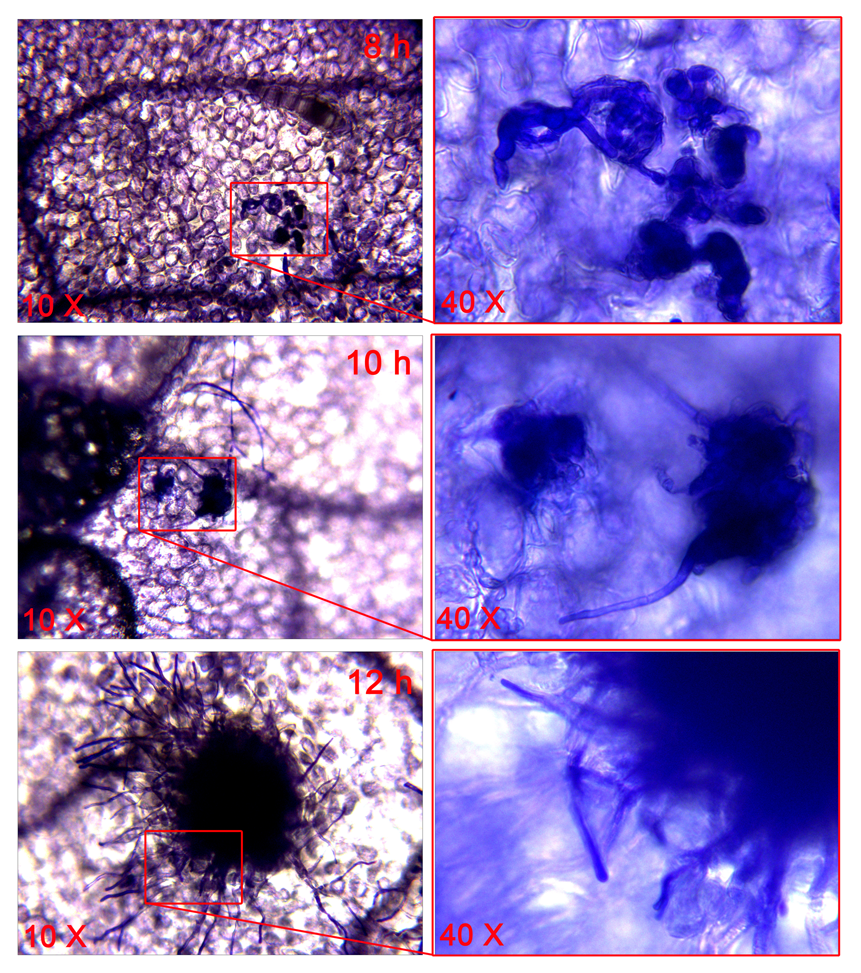

Supplement: S1 Fig — Leaves were stained with Coomassie Brilliant Blue R-250. The staining procedure was performed as described previously [87]. The images were captured with an Mshot CCD MS31. (TIF) [file ppat.1004878.s001.tif]

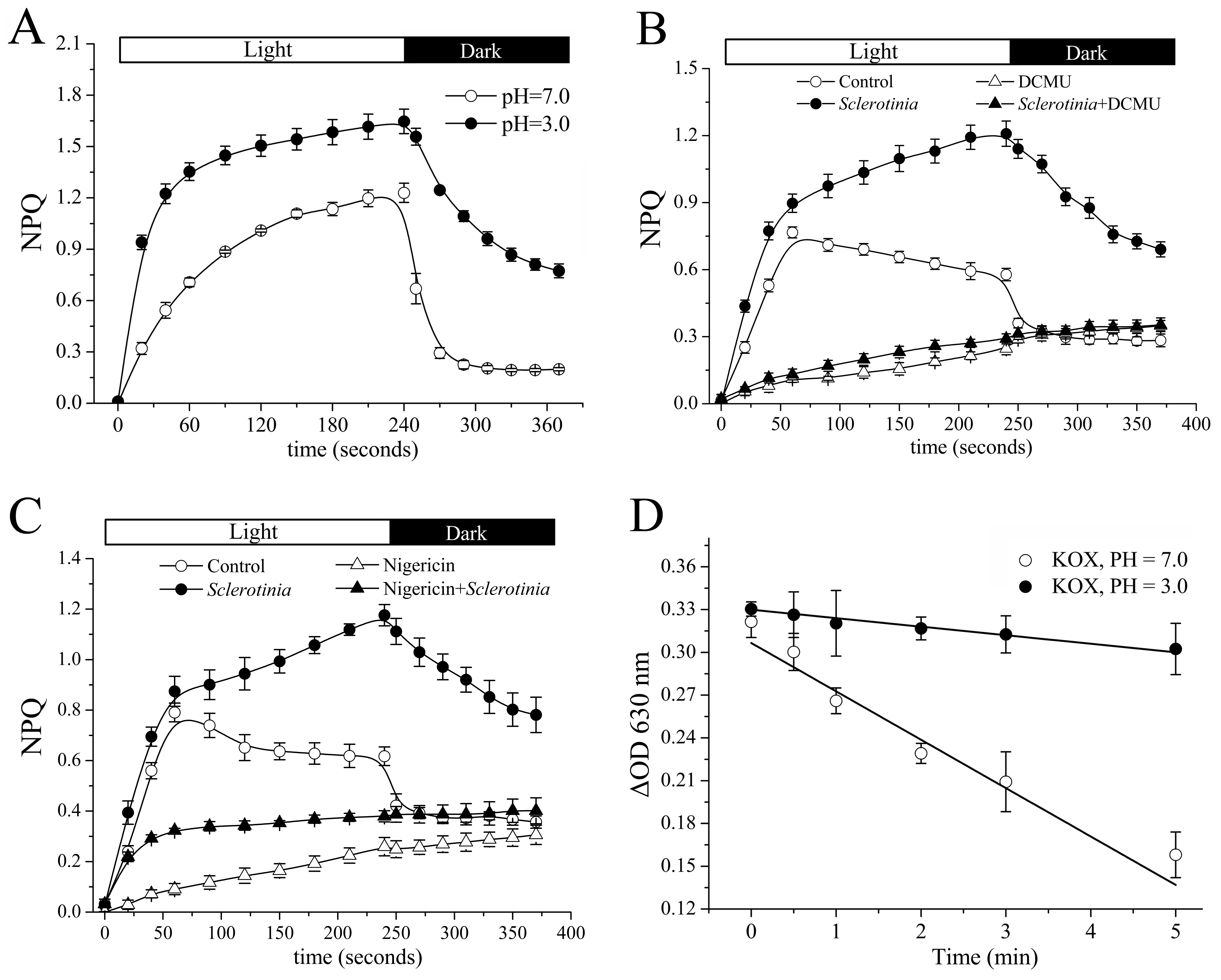

Supplement: S2 Fig — (A) Different kinetics of NPQ induced by KOX at pH 7.0 and 3.0. Detached leaves were syringe-infiltrated with 10 mM KOX buffered to 7.0 or 3.0 with HCl. (B, C) Impact of DCMU (B) and nigericin (C) on the kinetics of NPQ induced by Sclerotinia. Detached leaves were vacuum-infiltrated with DCMU (8 μM) or nigericin (50 μM). After inoculation with Sclerotinia, these leaves were dark-adapted for 1 h prior to measurement of NPQ. (D) KOX at pH 3.0 partially inhibited ATP synthase activity. After adding 4 mM ADP and 50 μM Pi, chloroplast suspensions were illuminated with 130 μmol photons m-2 s-1 for the indicated time. ATP synthase activity was monitored by colorimetric determination of the decrease in Pi at 630 nm. Values are means ± SE of three replicates. (TIF) [file ppat.1004878.s002.tif]

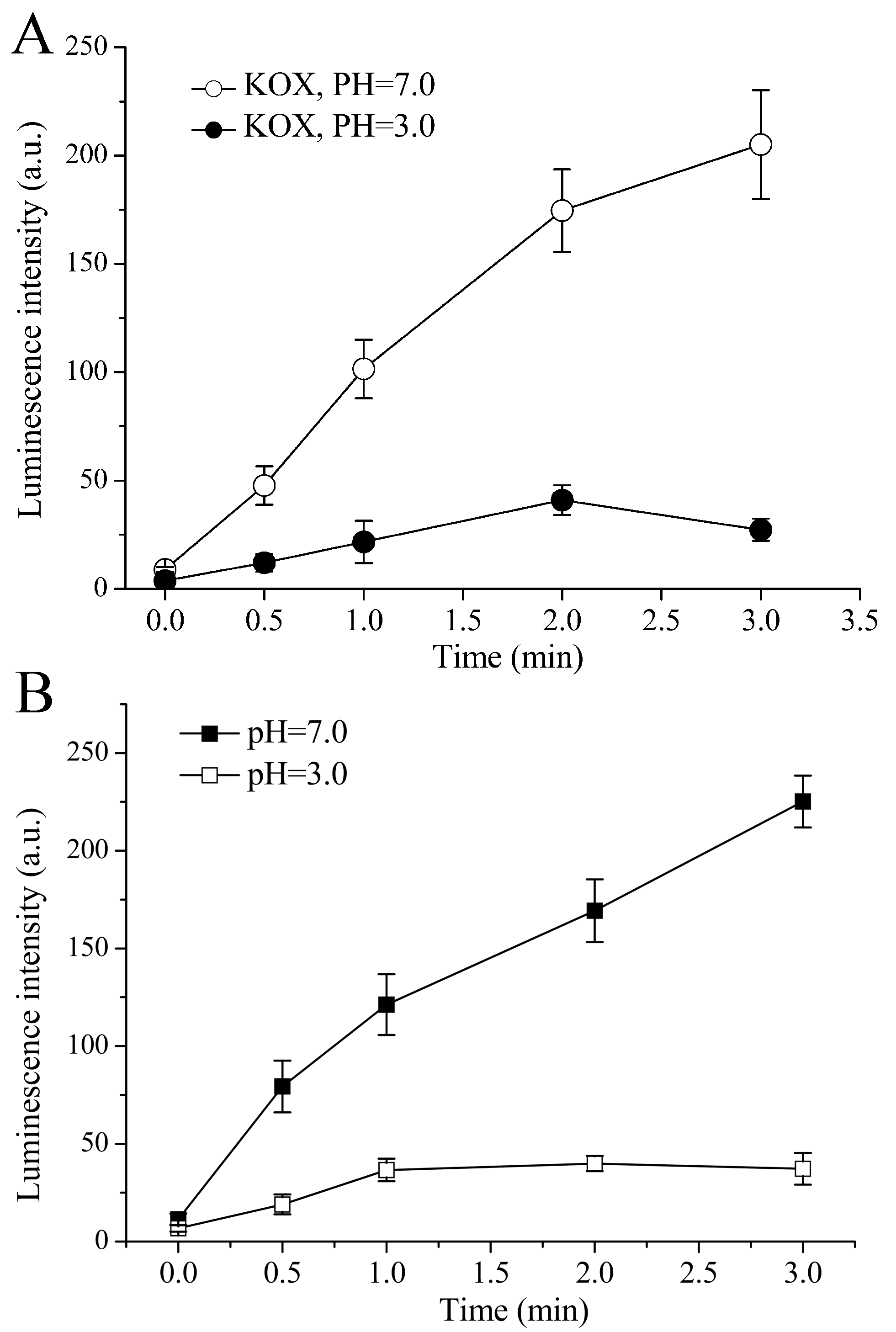

Supplement: S3 Fig — (A) KOX at pH 3.0 reduced the ATP synthase activity. (B) The effect of pH without KOX on ATP synthase activity. The ATP synthase activity were qualitatively measured by detecting ATP generation with firefly luciferin-luciferase reaction. After illumination with light (130 μmol photons m-2 s-1) for the indicated time (0–3 min), the solution was re-adjusted to pH 7.8 and added D-luciferin (100 μM) and luciferase (100 μg/mL). The luminescence was recorded by an ICCD (576S-1, Princeton) over a 5 min period. (TIF) [file ppat.1004878.s003.tif]

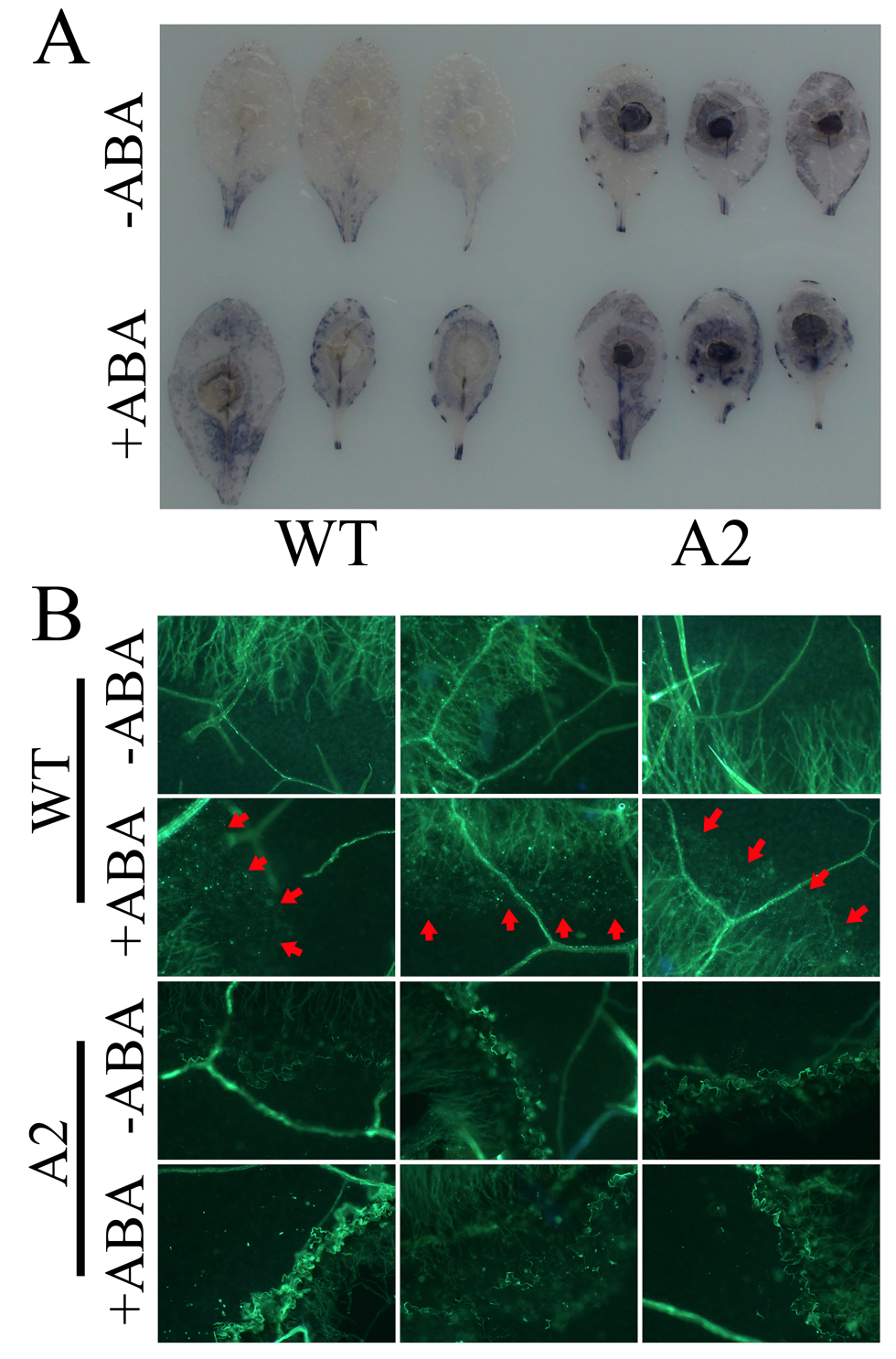

Supplement: S4 Fig — Pretreatment of ABA induced O2 - formation (A) and callose deposition (B) around the developing necrotic lesions. Arrows indicate the leading edge of callose deposition. NBT staining were photographed by a SONY numeric camera (HDR-XR500E). The images of callose deposition were captured with an Mshot CCD MS31. Three repetitions were given in each treatment groups. (TIF) [file ppat.1004878.s004.tif]

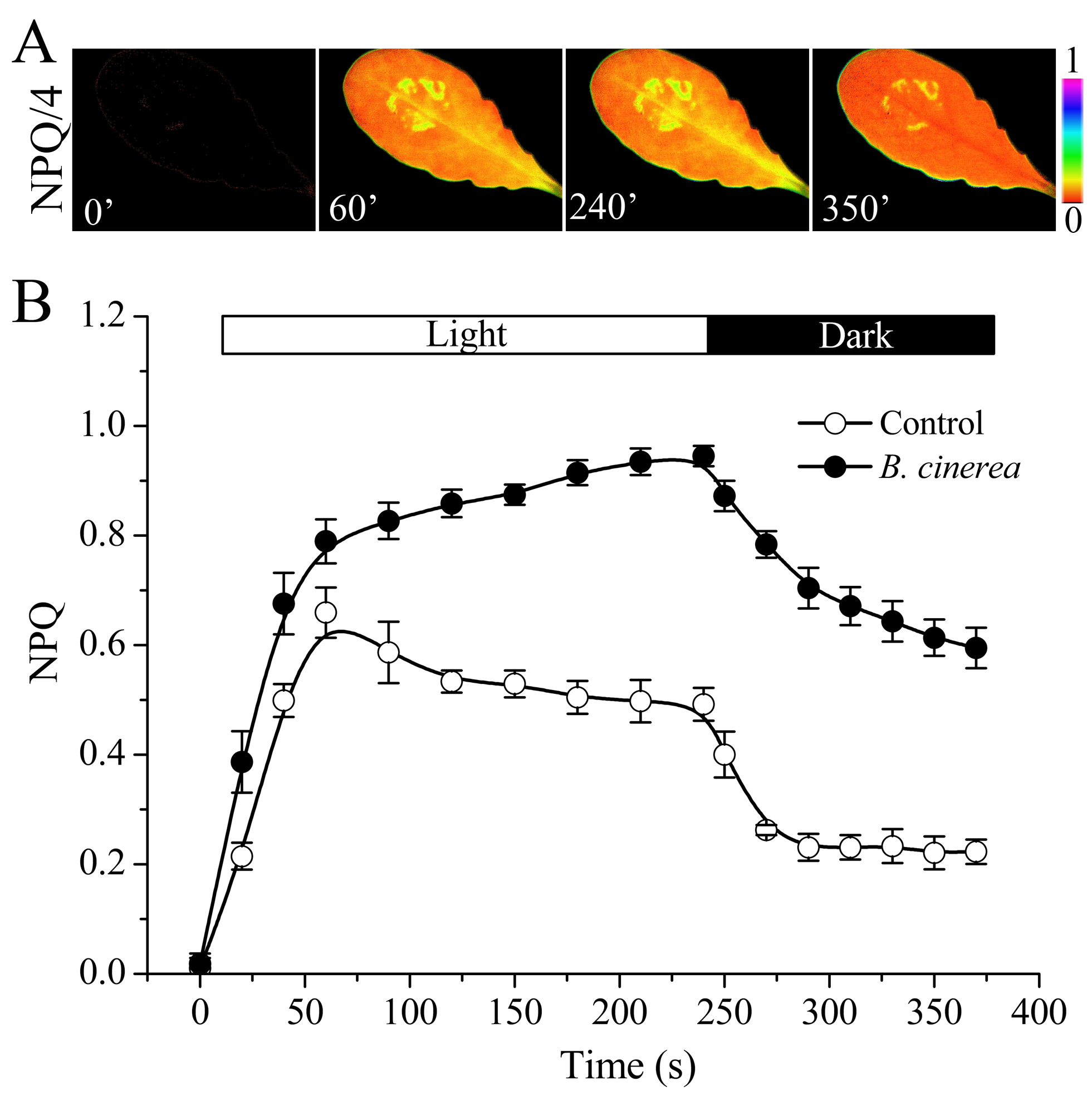

Supplement: S5 Fig — (A) Chlorophyll fluorescence image shows NPQ changes in B. cinema-infected Arabidopsis leaves. (B) Induction and relaxation kinetics of NPQ in B. cinema-infected zone. The light is switched off after 240 seconds. Each curve represents the average of three replicates ± SE. (TIF) [file ppat.1004878.s005.tif]

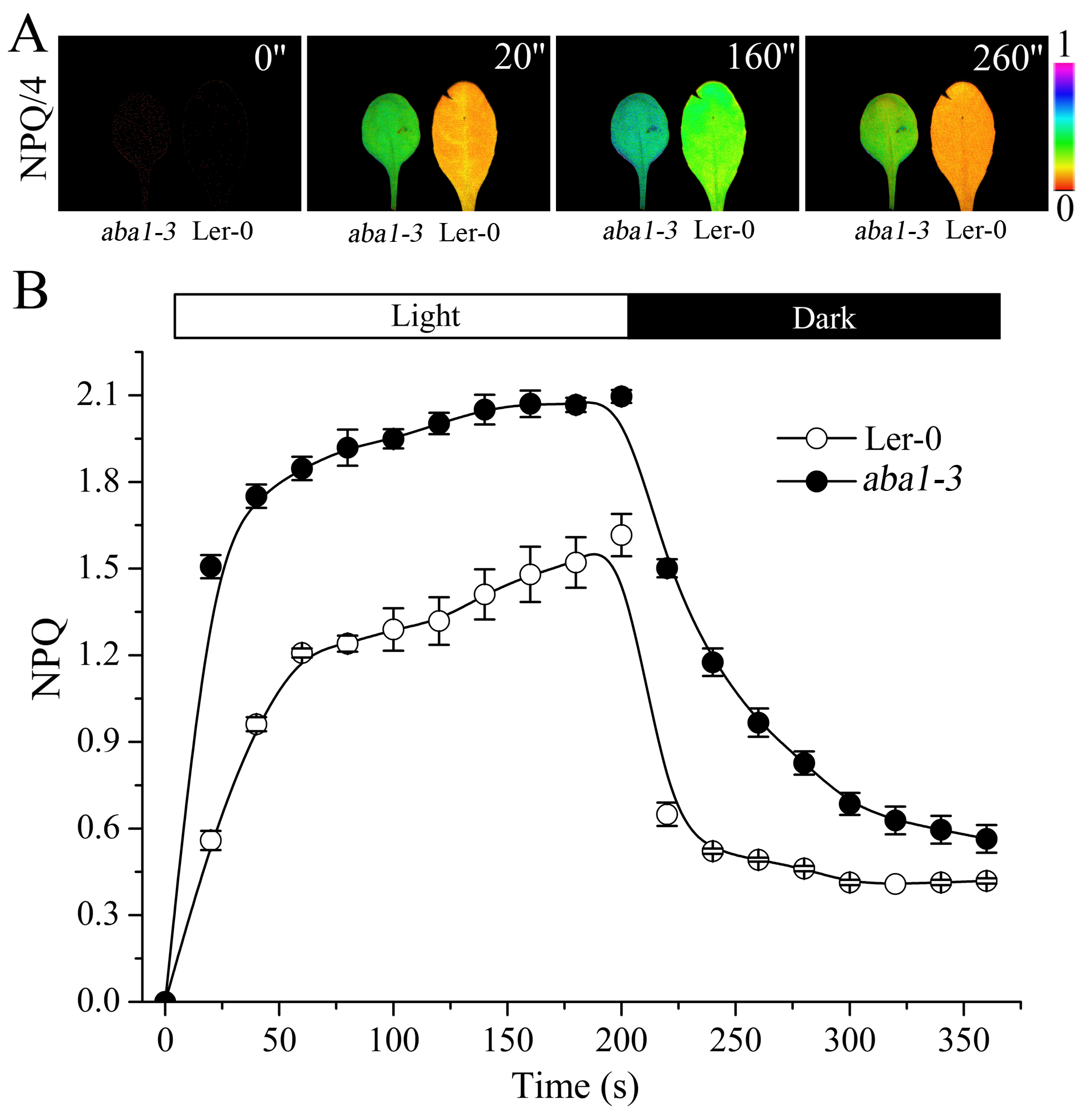

Supplement: S6 Fig — (A) Chlorophyll fluorescence image shows the difference of NPQ formation in aba1-3 and Ler-0 leaves. (B) Induction and relaxation kinetics of NPQ in aba1-3 and Ler-0 leaves. Actinic light of 725 μmol photons m-2 s-1 was selected to measure the dynamics of NPQ. The light is switched off after 200 seconds. Each curve represents the average of three replicates ± SE. (TIF) [file ppat.1004878.s006.tif]

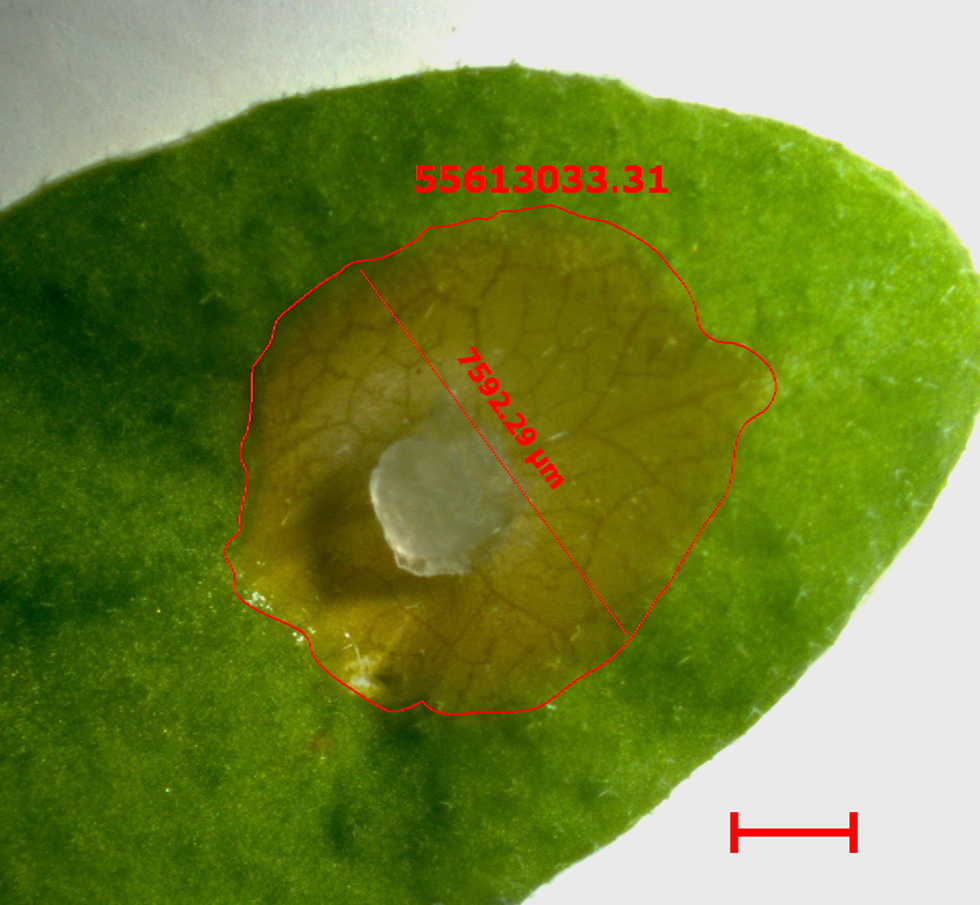

Supplement: S7 Fig — Photographs were initially captured using a Zeiss inverted microscope installed with a Carl Zeiss AxioCam MRc5 camera. Lesion area was quantified with the measurement tool ‘outline spline’ in AxioVision Rel.4.5 software. The unit of measurement is micrometer (μm). Bar = 2 mm. (TIF) [file ppat.1004878.s007.tif]

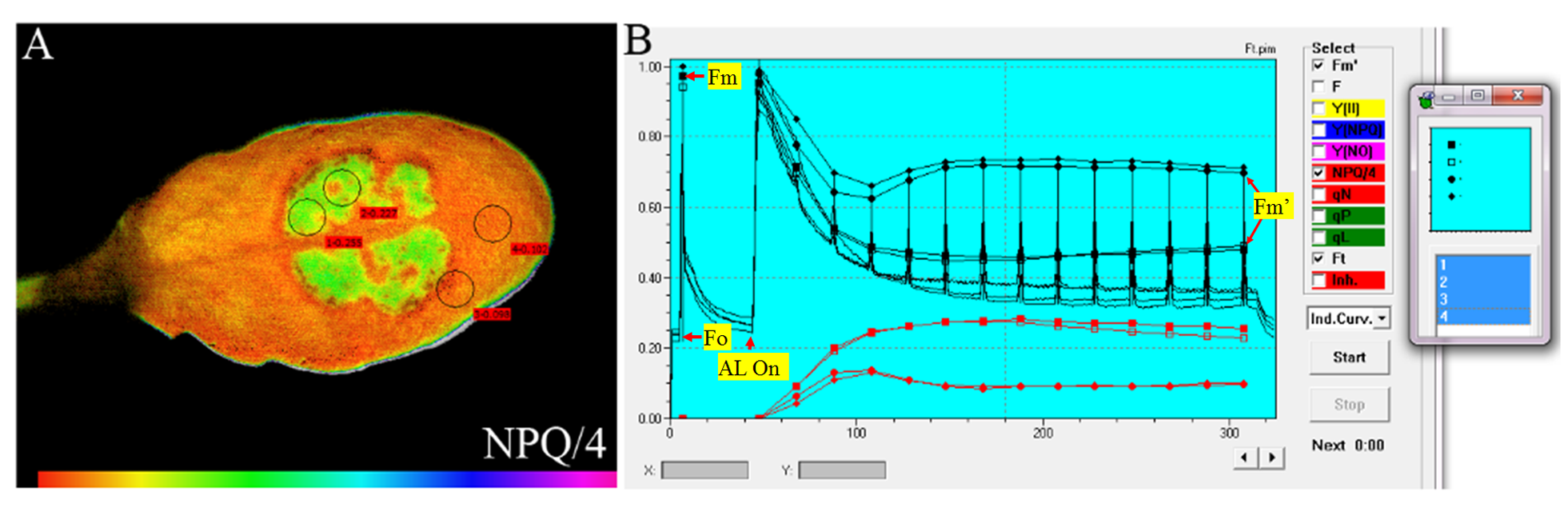

Supplement: S8 Fig — (A) Chlorophyll fluorescence image shows NPQ changes after infection with Sclerotinia. (B) The original trace of current fluorescence yield (Ft) in Sclerotinia-infected leaf. Because Ft is not continuously stored in the Buffer-Memory, a direct screenshot from the ImagingWin software was given. To facilitate comparison, the control (circles 3 and 4) here was selected from the un-inoculated region. Fo, Fm and Fm’ were marked in the Ft trace. “AL on” refer to turning the continuous actinic light (133 μmol photons m-2 s-1) on. The changes of NPQ/4 were showed below with red lines. (TIF) [file ppat.1004878.s008.tif]
