# Supplementary material for: Manipulation of the Xanthophyll Cycle Increases Plant Susceptibility to Sclerotinia sclerotiorum
Source: PLoS Pathog. 2015 May 20;11(5):e1004878. doi: 10.1371/journal.ppat.1004878 (PMC4439079; doi:10.1371/journal.ppat.1004878)
Supplement: S1 Table — (DOCX) [file ppat.1004878.s009.docx]

**Table S1.** **qPCR primers used for determining ABA biosynthesis genes.**

| Primer name | Sequence 5′-3′ |
| --- | --- |
| *NCED3-F* | CGGTGGTTTACGACAAGAACAA |
| *NCED3-R* | CAGAAGCAATCTGGAGCATCAA |
| *ABA1-F* | GGCATTTGGTC TAAGGTGAGAA |
| *ABA1-R* | CAGACTCGATATCCGCTGGTA |
| *ABA2-F* | TTCTCTTCCTAGTCAAAGGCTTT |
| *ABA2-R* | GCAGACTTTGGCACCGTGCT |
| *ABA3-F* | CAAAAGGAAGAGTCAAGAGGAAA |
| *ABA3-R* | TTTCTTTCATCAA CTTCACCAGAT |
| *AAO3-F* | GGAGTCAGCGAGGTGGAAGT |
| *AAO3-R* | TGCTCCTTCGGTCTGTCCTAA |
| *ACTIN2-F* | GTTGGGATGAACCAGAAGGA |
| *ACTIN2-R* | GAGGAGCCTCGGTAAGAAGA |
